# Efficacy and Safety of Pulsed‐Field Versus High‐Power Short‐Duration Ablation for Atrial Fibrillation: A Systematic Review and Meta‐Analysis With Reconstructed Time‐to‐Event Data

**DOI:** 10.1111/jce.16728

**Published:** 2025-05-28

**Authors:** Ahmed Mazen Amin, Mustafa Turkmani, Saman Al Barznji, Sanghamitra Mohanty, Rachel M. Kaplan, Jeffrey Winterfield, Dhanunjaya Lakkireddy, Pasquale Santangeli, Luigi Di Biase, Andrea Natale

**Affiliations:** ^1^ Faculty of Medicine Mansoura University Mansoura Egypt; ^2^ Faculty of Medicine Michigan State University, Faculty of Medicine East Lansing Michigan USA; ^3^ Department of Internal Medicine McLaren Health Care Oakland Michigan USA; ^4^ Texas Cardiac Arrhythmia Institute St. David's Medical Center Austin Texas USA; ^5^ Section of Cardiac Electrophysiology, Division of Cardiology Medical University of South Carolina (MUSC) Charleston South Carolina USA; ^6^ Kansas City Heart Rhythm Institute Overland Park Kansas USA; ^7^ Section of Cardiac Pacing and Electrophysiology Heart and Vascular Institute, Cleveland Clinic Cleveland Ohio USA; ^8^ Department of Electrophysiology Albert Einstein College of Medicine at Montefiore Health System New York New York USA; ^9^ Department of Biomedicine and Prevention, Division of Cardiology University of Tor Vergata Rome Italy

**Keywords:** atrial fibrillation, catheter ablation, high‐power short‐duration, pulmonary vein isolation, pulsed‐field ablation

## Abstract

**Background:**

Pulsed‐field ablation (PFA) and high‐power short‐duration (HPSD) ablation (45–90 W) are emerging technologies in atrial fibrillation (AF) treatment, both achieving durable pulmonary vein isolation. We aim to investigate the efficacy and safety of PFA versus HPSD ablation.

**Methods:**

We comprehensively searched PubMed, Web of Science (WOS), Scopus, EMBASE, and Cochrane Central Register of Controlled Trials (CENTRAL) through July 2024. Pairwise meta‐analysis with reconstructed time‐to‐event analysis were performed using R version 4.3.1 (PROSPERO ID: CRD42024576031).

**Results:**

Seven observational studies, including 1904 patients, were included. PFA was significantly associated with lower atrial tachyarrhythmia recurrence compared to HPSD ablation (45–90 W) at the longest follow‐up (RR: 0.73, 95% CI [0.60, 0.88], *p* < 0.01). Subgroup analysis revealed a significant reduction in atrial tachyarrhythmia recurrence with PFA versus HPSD ablation (45–50 W) (RR: 0.69, 95% CI [0.54, 0.88], *p* < 0.01), but not compared to vHPSD ablation (70–90 W). Reconnected pulmonary vein rates were significantly lower with PFA compared to HPSD (45–50 W) (*p* = 0.03), while no significant difference was observed compared to vHPSD (70–90 W). PFA was significantly associated with reduced procedural duration (MD: −33.15 with 95% CI [−40.93, −25.36], *p* < 0.01) and left atrial dwell time (MD: −32.16 with 95% CI [−45.55, −18.77], *p* < 0.01), although fluoroscopy time increased (MD: 7.48 with 95% CI [4.29, 10.68], *p* < 0.01) compared to HPSD ablation (45–90 W). Safety profiles were comparable, but pericarditis rates were significantly lower with PFA versus HPSD (45–50 W) (*p* = 0.003) and vHPSD (70–90 W) (*p* = 0.019). Kaplan–Meier analysis showed a 28% lower risk of atrial tachyarrhythmia recurrence with PFA compared to HPSD ablation (45–90 W) (HR: 0.72, 95% CI [0.57, 0.91], *p* = 0.006) over an 18‐month follow‐up.

**Conclusion:**

PFA and HPSD ablation (45–90 W) are effective and safe for AF ablation. PFA was significantly associated with lower atrial tachyarrhythmia recurrence, shorter procedural duration, reduced left atrial dwell time, increased fluoroscopy time, and comparable safety, with lower rates of pericarditis compared to HPSD ablation.

## Graphical abstract

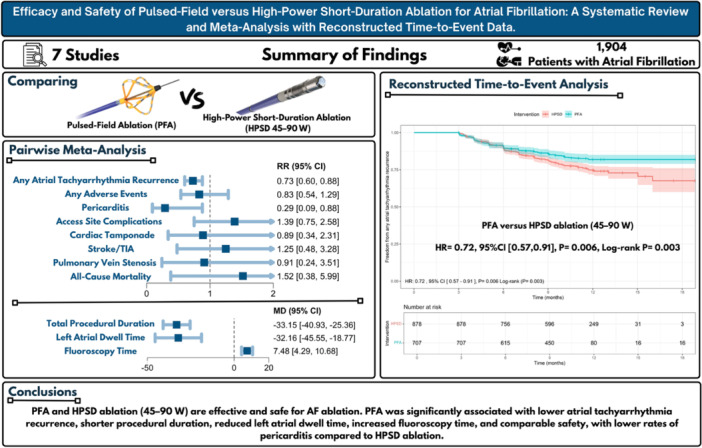

## 1 Introduction

Atrial fibrillation (AF) is the most prevalent heart rhythm disorder, leading to serious health complications and an increased risk of death [1]. The prevalence of AF has increased threefold over the past 50 years [2, 3]. In 2016, the Global Burden of Disease project estimated that approximately 46.3 million people worldwide lived with AF [4]. In the United States alone, 3–6 million individuals currently have AF, with projections suggesting this number could rise to 6–16 million by 2050 [5, 6].

Catheter ablation has emerged as an essential component in managing patients with AF. Recent international guidelines strongly recommend the procedure as a Class I indication in many clinical situations [7, 8, 9]. Pulmonary vein isolation (PVI) is the cornerstone of catheter ablation for managing symptomatic paroxysmal and persistent AF [7, 8].

Thermal ablation, whether using point‐by‐point radiofrequency or cryoballoon ablation, is the most common technique for PVI [10, 11]. However, ensuring safe and effective PVI with these methods can be challenging due to their lack of tissue selectivity and a high rate of pulmonary vein (PV) reconnection, with rates reported as high as 70% [12, 13, 14, 15, 16]. Additionally, both techniques carry a small but notable risk of collateral damage to nearby structures, such as the esophagus and phrenic nerve, and may also result in potential pulmonary vein stenosis [12, 13, 14, 15, 16].

Recently, high‐power short‐duration (HPSD) ablation and pulsed‐field ablation (PFA) have been introduced into clinical practice. Unlike conventional radiofrequency ablation, which typically operates at 20–40 W for 20‐40 s, HPSD ablation employs higher power levels (≥ 45 W) for a shorter duration (< 20 s). This approach has been shown to increase ablation lesions' volume and width/depth ratios. Consequently, these lesions tend to be shallower and adapt well to the thickness of the posterior wall, potentially providing extra protection to surrounding organs [17, 18, 19]. HPSD ablation is recognized for its safety profile, which is comparable to, or in some cases superior to, that of standard radiofrequency ablation while consistently reducing procedural times [18, 20, 21, 22, 23].

PFA is an innovative cardiac ablation technique that utilizes high‐voltage, ultrashort electrical pulses to form nanoscale pores in cardiac cell membranes through a process known as irreversible electroporation. This technique destabilizes the cell membrane, leading to leakage of cell contents and subsequent cell death, thereby creating transmural lesions. Unlike thermal ablation techniques, PFA disrupts cardiomyocyte function without heating the tissue, explicitly targeting cardiac tissue while preserving nearby structures such as blood vessels, nerves, and the esophagus [24, 25, 26, 27]. This technology has been shown to be associated with minimal risk of damage to nearby tissues, reduced risk of pulmonary vein stenosis, shorter procedure times, and greater efficacy than traditional thermal ablation methods [28, 29, 30, 31, 32].

Thus, this meta‐analysis aims to investigate the latest evidence on the efficacy and safety of PFA compared to HPSD ablation (45–90 W) for AF.

## 2 Methodology

### 2.1 Protocol Registration

We conducted this meta‐analysis following the PRISMA statement guidelines for systematic reviews and Meta‐Analyses [33] and the Cochrane Handbook for Systematic Reviews and Meta‐analysis guidelines [34]. It was prospectively registered in the International Prospective Register of Systematic Reviews (PROSPERO) under ID: CRD42024576031.

### 2.2 Data Sources and Search Strategy

We searched the following databases comprehensively: PubMed, Cochrane Central Register of Controlled Trials (CENTRAL), Scopus, Web of Science, and EMBASE up to July 2024 using the keywords “Pulsed‐field” AND “High‐power shorter duration” in “Atrial fibrillation”. Further details are delineated in Supporting Information S1: Table S1.

### 2.3 Eligibility Criteria

In the pairwise meta‐analysis model, we included any comparative study that met our PICO. The population (*P*) consists of patients with AF, regardless of the type of AF or whether it is a first or repeat ablation. Intervention (I) is PFA. The control (C) is HPSD ablation (45–90 W). The outcomes (O) encompassed the primary outcomes: any atrial tachyarrhythmia (ATA) recurrence and PVs reconnection; secondary outcomes: total procedural duration, left atrial dwell time, and fluoroscopy time; and safety outcomes: any adverse events (AEs), access site complications, cardiac tamponade, pericarditis, stroke/transient ischemic attack (TIA), pulmonary vein stenosis, and all‐cause mortality.

In the reconstructed time‐to‐event model, we included any comparative study with published Kaplan‐Meier survival curves comparing PFA versus HPSD ablation (45–90 W) for patients with AF.

### 2.4 Study Selection

After searching the databases and retrieving records, duplicates were removed using Covidence online software. Three reviewers (A.M.A., M.T., and S.A.) independently screened the titles and abstracts of the relevant records, followed by the full texts according to the previously mentioned eligibility criteria. Any conflicts were resolved through discussion.

### 2.5 Data Extraction

Three reviewers (A.M.A., M.T., and S.A.) independently extracted data from the included studies using an Excel sheet encompassing; (1) a summary sheet (study design, country, the total number of patients with AF, AF type included, PFA procedure details (access, sheath used, mapping system if used, and the procedure technique), HPSD ablation procedure details (access, sheath used, mapping system if used, power used, and the procedure technique), first or repeat ablations, additional ablations, blanking period, ATA recurrence definition and monitoring, main inclusion criteria, primary outcome, and follow‐up duration); (2) baseline information (number of patients with AF in PFA and HPSD ablation groups, male sex percentage, age (years), body mass index (BMI), CHA_2_DS_2‐_VASc score, left ventricular ejection fraction, AF type (paroxysmal and persistent), comorbidities (hypertension, diabetes, coronary artery disease, and stroke/TIA); (3) outcomes sheet (ATA recurrence, PVs reconnection, total procedural duration, left atrial dwell time, fluoroscopy time, any AEs, access site complications, cardiac tamponade, pericarditis, stroke/TIA, pulmonary vein stenosis, and all‐cause mortality). Conflicts were discussed and resolved by consensus.

### 2.6 Risk of Bias

Three reviewers (A.M.A., M.T., and S.A.) used the Cochrane Risk Of Bias In Non‐randomised Studies—of Interventions (ROBINS‐I) tool to assess the quality of non‐randomized comparative studies [35]. This tool has seven domains to evaluate the bias arising from the existence of the confounding variables, the selection of the participants, the classification of the study groups, deviations from the protocol, missing outcomes, measurement of the outcomes, and selection of the reported results.

### 2.7 Statistical Analysis

Our statistical analysis was conducted using R Statistical Software (version 4.3.1, Foundation for Statistical Computing, Vienna, Austria). Regarding the pairwise meta‐analysis model, we pooled our outcomes using the risk ratio (RR) with a 95% confidence interval (CI). We used the random‐effects model when significant heterogeneity was present (*I* ^2^ > 50%) and the fixed‐effect model when heterogeneity was less (*I* ^2^ < 50%). We used the *χ* ^2^ and I‐square statistic to assess the heterogeneity, where the *χ* ^2^ test assesses the presence of heterogeneity, and the I‐square statistic assesses its degree. We considered an alpha level below 0.1 for the *χ* ^2^ test to denote significant heterogeneity. In case of high heterogeneity, we conducted a leave‐one‐out sensitivity analysis, in which we omitted each study in one scenario to detect the source of the heterogeneity. We conducted a subgroup analysis for the HPSD ablation arm (45–90 W) based on the different power levels: HPSD ablation (45–50 W), very high‐power short‐duration (vHPSD) ablation (70 W), and vHPSD ablation (90 W). Additionally, we performed a meta‐regression to assess how baseline characteristics influenced the RR of ATA recurrence. Funnel plots and Egger's test were used to detect publication bias across all outcomes in the included studies.

In the reconstructed time‐to‐event data analysis, we reconstructed individual patient data from the published Kaplan–Meier curves of all the included studies utilizing the curve approach [36]. We adopted the two‐stage approach described by Liu et al. [37], adopting the “IPDfromKM” R package. First, we extracted raw data coordinates (time, survival probability) of each arm of the included Kaplan–Meier curves. Then, individual patient data were reconstructed based on the raw data coordinates and the number of patients at risk at reported time points. Finally, we merged the reconstructed time‐to‐event data of all individual studies in a merged data set. We used the log‐rank test to investigate the difference in freedom from ATA recurrence between the two groups.

We used the Cox frailty regression model to calculate the hazard ratio (HR) with a 95% CI for the difference between PFA and HPSD ablation (45–90 W) and included the γ frailty term to assess the between‐studies heterogeneity, where individual studies are modelled as a random effect. Then, we used the likelihood ratio test to test the significance of this γ frailty term. Considering the different blanking periods in the studies, we considered the first 3 months as the blanking period for all studies. Additionally, we conducted a further sub‐grouped reconstructed time‐to‐event data analysis for the HPSD ablation arm (45–90 W) based on the different power levels (HPSD ablation (45–50 W) and vHPSD ablation (70–90 W)). A further sensitivity analysis was also conducted, considering only the first month as the blanking period.

Moreover, we tested the proportional hazards assumption using the Grambsch‐Therneau test, log‐log survival curve, and diagnostic plots based on Schoenfeld residuals [38], and we employed a robust variance estimator to accommodate violations of the assumption of homoscedasticity, which assumes equal or similar variances across different groups being compared. We conducted a landmark analysis in the case of violating the proportional hazards assumption. We calculated Flexible parametric survival models with B‐splines to provide HRs with 95% CI of the association between PFA versus HPSD ablation (45–90 W) and ATA recurrence, allowing a time‐varying effect [39]. Finally, using the R package “survRM2”, we analyzed the variation in restricted mean survival times (RMSTs) over time [40]. Furthermore, we employed the *χ* ^2^ test to investigate the statistical significance of the differences in the number of patients with reconnected PVs, the number of reconnected PVs, and the safety profile. A Jackknife resampling analysis was performed on the overall population by systematically excluding one study at a time. A *p* value of less than 0.05 was considered statistically significant.

## 3 Results

### 3.1 Search Results and Study Selection

After searching the following databases: PubMed, CENTRAL, Scopus, Web of Science, and EMBASE, we reached 81 records. Covidence detected and removed 38 duplicates and found 43 records eligible for title and abstract screening. We excluded 19 irrelevant studies and found 24 records eligible for full‐text screening. Finally, we included seven studies (Supporting Information: Figure S1).

### 3.2 Characteristics of Included Studies

We included seven studies [41, 42, 43, 44, 45, 46, 47] with a total of 1904 patients: 763 in the PFA arm, 1141 in the HPSD ablation (45–90 W) arm (620 in the HPSD ablation (45–50 W) arm, 117 in the vHPSD ablation (70 W) arm, and 404 in the vHPSD ablation (90 W) arm. Specifically, 1563 patients had paroxysmal AF, and 341 had persistent AF. All studies utilized the Farapulse system. More details about the characteristics of included studies, included patients, and procedural details are outlined in Tables 1, –, 3. Further details for the PFA and HPSD ablation procedures (access, sheath used, mapping system if used, and the procedure technique) are outlined in Supporting Information S1: Tables S2 and S3. Moreover, only Russo et al. [47] and Della Rocca et al. [42] employed propensity score matching (PSM) to account for the confounding variables that existed between the PFA and HPSD ablation groups. More details about the matching criteria and the variables used in each model are detailed in Supporting Information S1: Table S4.

**Table 1:** Summary characteristics of the included studies.

Study ID	Study design	Country	Total participants	Paroxysmal or persistent AF	First or redo ablation	PFA	HPSD ablation			Additional ablation	Blanking period	Main inclusion criteria	ATA recurrence definition and monitoring	Primary outcome	Follow‐up duration
							Power	Catheter	Mapping system						
Badertscher et al. [41]	Single‐center, prospective cohort study	Switzerland	115	Paroxysmal and persistent AF	First ablation	FaraWave catheter (Farapulse)	50 W	Irrigated‐tip ThermoCool catheter	No mapping system	NA	No blanking period	Patients undergoing their first PVI	NA	AF recurrence	Median [IQR]: 214 [107–380] days.
Della Rocca et al. [42]	Multi‐center, retrospective cohort study	Belgium, USA, France, and Italy	522	Paroxysmal AF	First ablation	Pentaspline Farawave catheter (Farapulse)	45 W	Open‐irrigated RF ablation catheter (Thermocool)	CARTO 3 (Biosense Webster) and multipolar catheter (Pentaray)	PVI‐only ablation	3 months	Patients undergoing first‐time catheter ablation of non‐valvular paroxysmal AF	Definition: any atrial tachyarrhythmia > 30 s off AADs and occurring after the 3‐month blanking period. Monitoring: Each visit included a physical examination, 12‐lead ECG, and 24‐h Holter monitoring. Alternatively, 7‐day Holter monitoring was prescribed in the presence of symptoms suggestive of arrhythmia recurrence. AADs were resumed before hospital discharge, maintained for the first 3 months postablation (blanking period), and discontinued thereafter if no arrhythmic recurrences were documented during this period.	ATA recurrence	Mean (SD): 12.3 (2.3) months.
Popa et al. [43]	Single‐center, retrospective cohort study	Germany	127	Paroxysmal AF	First ablation	Pentaspline Farawave catheter (Farapulse)	vHPSD (70 W): 70 W/7 s (AW) or 70 W/5 s (PW), vHPSD (90 W): 90 W/4 s (AW and PW)	vHPSD (70 W): irrigated‐tip catheter FlexAbility SE and the Ampere RF generator. vHPSD (90 W): irrigated‐tip catheter QDOT MICRO and the nGEN RF generator	vHPSD (70 W): EnSite Precision and the Advisor circular catheter (Abbott): vHPSD (90 W): The CARTO 3 system and the Lasso catheter (BiosenseWebster)	PVI‐only ablation	2‐months	Paroxysmal AF receiving first‐time PVI	Definition: any atrial arrhythmia (AF or atrial tachycardia) with a duration ≥ 30 s documented after a 2‐month blanking period off AADs following the ablation procedure. Monitoring: a 12‐lead electrocardiogram (ECG), a 5‐day Holter ECG, and a review of any other available external ECGs.	Quantify differences in myocardial injury and inflammation	6 months
Reinsch et al. (PRIORI Study) [46]	Single‐center, retrospective cohort study	Germany	410	Paroxysmal AF	First ablation	Pentaspline PFA catheter (Farawave, Farapulse)	45 W	Thermocool Smarttouch SF Catheter	CARTO 3 (Biosense Webster) and pentaspline mapping catheter (Pentaray, Biosense Webster)	No additional ablations outside of the PV were applied	3 months	First time PVI for paroxysmal AF	Definition: any atrial tachyarrhythmias lasting more than 30 s identified on surface ECG or Holter monitoring, off AADs. Monitoring: Clinical and 5‐day Holter ECG follow‐up. Any AADs, with the exception of ß‐blocker, were stopped 3 months after the initial ablation procedure if acceptable.	ATA recurrence	1 year
Russo et al. [47]	Multi‐center, retrospective cohort study	Italy	534	Paroxysmal and persistent AF	First and redo ablation	Farapulse system	90 W/4 s	QDM RF catheter	Electroanatomic mapping systems were used, but the system is NA	Additional structures beyond the pulmonary veins were ablated in approximately one‐third of the study cohort	1 month	Patients with symptomatic paroxysmal or persistent AF undergoing a first or redo AF catheter ablation	Definition: any atrial tachyarrhythmia (AF, atrial flutter, and atrial tachycardia) recurrence ≥ 30 s during follow‐up after a 1‐month blanking period. Monitoring: Through 24‐h Holter monitoring, 12‐lead electrocardiography, and clinical controls. At the last follow‐up, 26% of the overall study population was receiving Class I or III AADs.	ATA recurrence	Median [IQR]: 12 [9– 2] months
Soubh et al. [45]	Single‐center, retrospective cohort study	Germany	82	Paroxysmal and persistent AF	First ablation	Farapulse system	90 W/4 s	QDOT MICRO catheter (Biosense Webster, USA)	CARTO 3 System (Biosense Webster, USA) with Pentaray Nav ECO mapping	Right atrial ablations	3 months	Patients with symptomatic AF underwent PVI using PFA or vHPSD‐RF	Definition: Arrhythmia‐free survival. Monitoring: clinical assessment, ECG Holter monitoring for at least 3 days.	Arrhythmia‐free survival	6 months
Wörmann et al. [44]	Single‐center, retrospective cohort study	Germany	114	Paroxysmal and persistent AF	First ablation	Farapulse system	70 W for 7 s was used at all sites except for the posterior wall, where RF duration was reduced to 5 s	A noncontact‐force ablation catheter with an enhanced tip irrigation (20 mL/min) and distally positioned thermocouples (Flexibility D‐ or F‐Curve; Abbott)	(Ensite Precision or Ensite X; Abbott)	All patients, irrespective of their AF history (paroxysmal or persistent AF), received PVI only without additional ablation of atrial substrate or anatomical lines	3 months	Patients undergoing de novo PFA‐PVI for symptomatic paroxysmal or persistent AF	Definition: Any detected atrial arrhythmia (AF, atrial tachycardia, atrial flutter) longer than 30 s was defined as a recurrence of arrhythmia after a 90‐day blanking period. Monitoring: photopletysmogram (PPG) app‐based tele‐consultation (Fibricheck), 48 h Holter, and CIED interrogation if applicable. If abnormal PPGs were encountered, patients underwent a 24‐h Holter ECG or a 12‐lead ECG to ensure relapse of AF. AADs are discontinued immediately after PVI in patients suffering from paroxysmal AF and after 3 months in patients in the setting of persistent AF. At follow‐up, 3 patients (5%) in the PFA group and 5 patients (9%) in the HPSD group were on antiarrhythmic drugs; all suffered persistent AF.	Arrhythmia‐free survival	Median [IQR]: 125 [109–162] days

Abbreviations: AADs, anti‐arrhythmicantiarrhythmic drugs; AF, atrial fibrillation; ATA, atrial tachyarrhythmia; AW, anterior wall; ECG, electrocardiogram; HPSD, high‐power short duration; IQR, interquartile range; NA, not available; PFA, pulsed‐field ablation; PVI, pulmonary vein isolation; PW, posterior wall; RF, radiofrequency; SD, standard deviation.

**Table 2:** Baseline characteristics of the participants.

Study	Number of patients in each group		Age (years), mean (SD)		Male		BMI, mean (SD)		CHA_2_DS_2_VASc, Mean (SD)		LVEF, mean (SD)		LAD, Mean (SD)		AF type *N*. (%)				Comorbidities *N*. (%)							
	PFA	HPSD	PFA	HPSD	PFA	HPSD	PFA	HPSD	PFA	HPSD	PFA	HPSD	PFA	HPSD	Paroxysmal		Persistent		HTN		DM		CAD		Stroke/TIA	
															PFA	HPSD	PFA	HPSD	PFA	HPSD	PFA	HPSD	PFA	HPSD	PFA	HPSD
Badertscher et al. [41]	52	63	65 (10)		NA	NA	NA	NA	NA	NA	56 (11)		41 (6.6)		64 (56)		51 (44)		NA	NA	NA	NA	NA	NA	NA	NA
Della Rocca et al. [42]	174	348	62 (11.6)	62.9 (10.1)	110 (63.2)	217 (62.4)	27 (4.8)	25.8 (6.7)	2 (1.5)	2 (1.5)	59.4 (4.2)	57.9 (6.9)	41.8 (4.9)	41.7 (4.4)	174 (100)	348 (100)	0 (0)	0 (0)	78 (44.8)	146 (42)	16 (9.2)	34 (9.8)	10 (5.7)	22 (6.3)	8 (4.6)	14 (4.0)
Popa et al. (PFA vs. vHPSD (70 W) [43]	35	60	61.6 (11.4)	64.2 (9.2)	24 (68.6)	30 (50)	26.7 (4.9)	26.8 (4.6)	1.7 (1.7)	2.0 (1.7)	58.5 (2.7)	59.2 (4.7)	NA	NA	35 (100)	60 (100)	0 (0)	0 (0)	14 (40.0)	36 (60)	1 (2.9)	3 (5)	NA	NA	4 (11.4)	4 (6.7)
Popa et al. (PFA vs. vHPSD (90 W) [43]	35	32	61.6 (11.4)	62.2 (12.8)	24 (68.6)	20 (62.5)	26.7 (4.9)	25.4 (2.7)	1.7 (1.7)	2.0 (1.4)	58.5 (2.7)	57.9 (7.8)	NA	NA	35 (100)	32 (100)	0 (0)	0 (0)	14 (40.0)	16 (50)	1 (2.9)	3 (9.4)	NA	NA	4 (11.4)	2 (6.3)
Reinsch e al. (PRIORI Study) [46]	201	209	66.7 (10.5)	67.3 (11.9)	114 (56)	111 (53)	27.3 (3.7)	27.6 (4.5)	2.3 (2.2)	2 (1.5)	NA	NA	NA	NA	201 (100)	209 (100)	0 (0)	0 (0)	133 (66)	141 (67)	22 (11)	27 (13)	19 (9)	21 (10)	11 (5)	22 (11)
Russo et al. (Before PSM) [47]	192	342	65 (7.5)	62.7 (9.7)	121 (63)	223 (65)	25.2 (1.1)	25.2 (1.1)	2 (1.5)	1.7 (0.7)	57.7 (5.2)	58.3 (3.7)	NA	NA	130 (68)	238 (70)	62 (32)	104 (30)	121 (63)	204 (60)	56 (29)	41 (12)	26 (14)	54 (16)	NA	NA
Russo et al. (After PSM) [47]	171	171	64.3 (7.5)	65.3 (9.0)	107 (63)	111 (65)	25.2 (1.1)	25.3 (1.4)	2 (1.5)	2 (1.5)	57.7 (5.2)	58.3 (3.7)	NA	NA	115 (67)	117 (68)	56 (33)	54 (32)	105 (61)	106 (62)	39 (23)	34 (20)	24 (14)	22 (13)	NA	NA
Soubh et al. [45]	52	30	67 (10)	73 (7)	37 (71)	14 (47)	28 (6)	27 (6)	4 (1.6)	4 (1.7)	52 (8)	47 (14)	NA	NA	25 (48)	13 (43)	27 (52)	17 (57)	43 (83)	25 (83)	8 (15)	4 (13)	9 (17)	13 (43)	5 (10)	4 (13)
Wörmann et al. [44]	57	57	67 (13)	67 (12)	19 (33)	23 (40)	28 (5)	27 (4)	NA	NA	56 (6)	56 (9)	39.6 (6.0)	38.0 (3.5)	17 (30)	17 (30)	40 (70)	40 (70)	37 (65)	34 (60)	9 (16)	8 (14)	14 (25)	11 (19)	NA	NA

Abbreviations: AF, atrial fibrillation; BMI, body mass index; CAD, coronary artery disease; DM, diabetes mellitus; HPSD, high‐power short duration; HTN, hypertension; LAD, left atrium diameter; LVEF, left ventricular ejection fraction; NA, not available; PFA, pulsed‐field ablation; PSM, propensity score matching; SD, standard deviation; TIA, transient ischemic attack.

**Table 3:** Procedural details of pulsed‐field ablation and high‐power short‐duration catheter ablation.

Study	Patients with additional ablations		Acute reconnections		
	PFA, *N*. (%)	HPSD, *N*. (%)	PFA, *N*. (%)	HPSD, *N*. (%)	How was it assessed?
Badertscher et al. [41]	NA	NA	NA	NA	NA
Della Rocca et al. [42] (After PSM)	PVI‐only ablation	PVI‐only ablation	NA	NA	NA
Popa et al. [43]	PVI‐only ablation	PVI‐only ablation	0 (0)	40 (44.9)	Rechecking the entrance block after ≥ 20 min or adenosine administration
Reinsch e al. (PRIORI Study) [46]	No additional ablations outside of the PV were applied	No additional ablations outside of the PV were applied	NA	NA	NA
Russo et al.[47] (Unmatched populations)	PVI plus: 70 (36)	PVI plus: 118 (35)	NA	NA	NA
	PW: 67 (35)	PW: 71 (21)			
	CTI line: 8 (1)	CTI line: 50 (15)			
	LAA:1 (0.5)	LAA: 10 (3)			
	CS: 0 (0)	CS: 15 (4)			
	Mitral Isthmus: 0 (0)	Mitral Isthmus: 5 (1)			
	Left atrial roof line: 0 (0)	Left atrial roof line: 6 (2)			
	Superior cava vein: 0 (0)	Superior cava vein: 3 (1)			
Russo et al. [47] (Matched populations)	PVI plus: 64 (37)	PVI plus: 63 (37)	NA	NA	NA
	PW: 61 (36)	PW: 40 (23)			
	CTI line: 8 (5)	CTI line: 24 (14)			
	LAA:1 (1)	LAA: 7 (4)			
	CS: 0 (0)	CS: 11 (6)			
	Mitral Isthmus: 0 (0)	Mitral Isthmus: 2 (1)			
	Left atrial roof line: 0 (0)	Left atrial roof line: 4 (2)			
	Superior cava vein: 0 (0)	Superior cava vein: 1 (1)			
Soubh et al. [45]	CTI: 1 (2)	CTI: 13 (43) LA ablation lines: 9 (30)	NA	NA	NA
Wörmann et al. [44]	All patients, irrespective of their AF history (paroxysmal or persistent AF), received PVI only without additional ablation of atrial substrate or anatomical lines.	All patients, irrespective of their AF history (paroxysmal or persistent AF), received PVI only without additional ablation of atrial substrate or anatomical lines.	NA	NA	NA

Abbreviations: CS, coronary sinus; CTI, cavotricuspid isthmus; HPSD, high‐power short‐duration; LAA, left atrial appendage; NA, not available; PFA, pulsed‐field ablation; PV, pulmonary vein; PVI, pulmonary vein isolation; PW, posterior wall.

### 3.3 Risk of Bias

Upon assessing the risk of bias by ROBINS‐I, four studies showed a low risk of bias [42, 43, 44, 46], and three showed moderate concerns due to bias arising from the confounding variables [41, 45, 47], as outlined in Supporting Information S1: Figure S2.

### 3.4 Primary Outcomes (Any Atrial Tachyarrhythmia Recurrence and Pulmonary Veins Reconnection)

**Figure 1:**
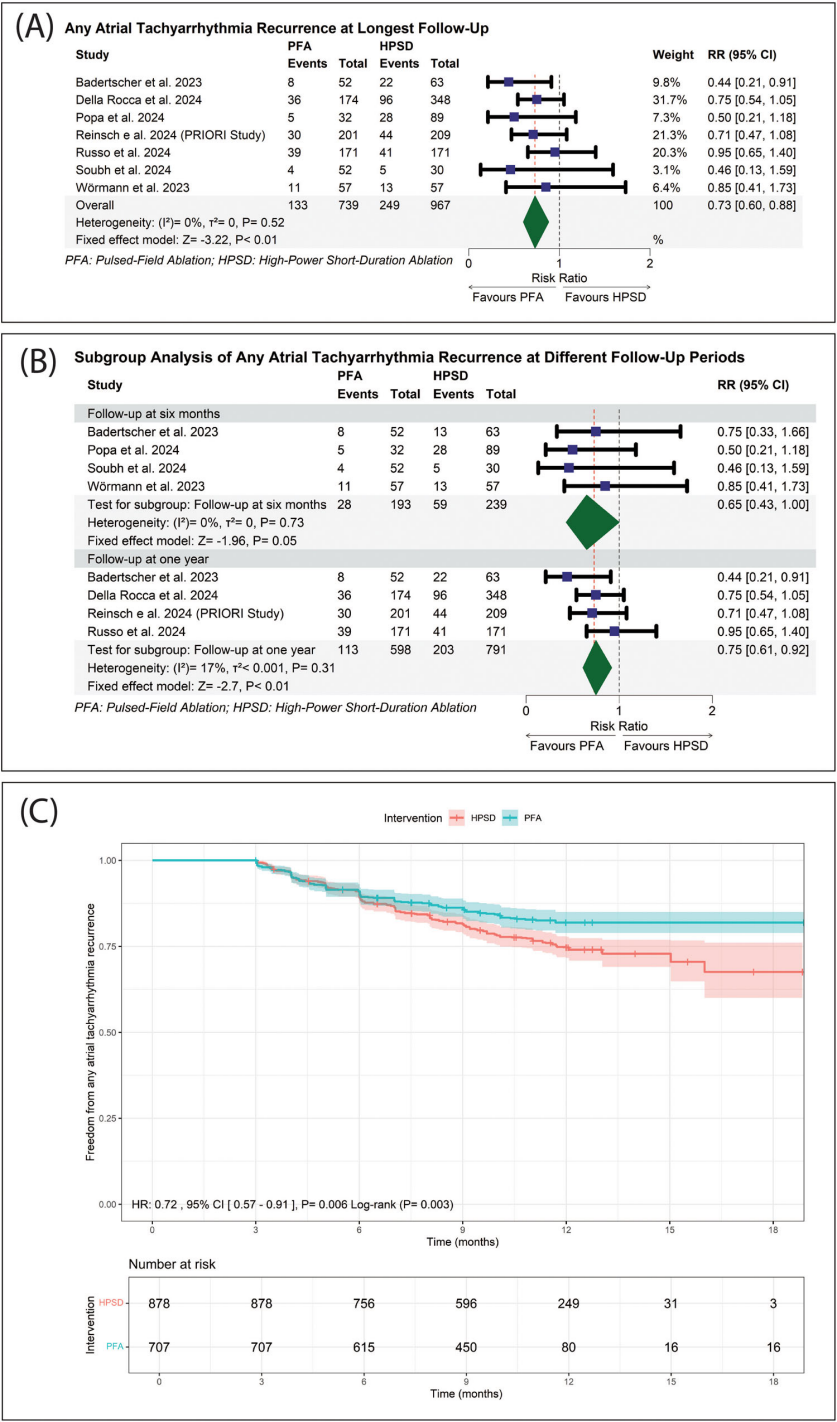
(A) Forest plot of pooled any atrial tachyarrhythmia recurrence at longest follow‐up. (B) Forest plot of any atrial tachyarrhythmia recurrence at 6 months and 1 year of follow‐up. (C) Pooled Kaplan–Meier curve showing the freedom from any atrial tachyarrhythmia recurrence. HR, hazard ratio.

PFA was significantly associated with a 27% reduction in ATA recurrence compared to HPSD ablation (45–90 W) at the longest follow‐up in each study (RR: 0.73 with 95% CI [0.60, 0.88], *p* < 0.01) (Figure 1A). Pooled studies were homogenous (*I* ^2^ = 0%, *p* = 0.52).

However, upon subgrouping the studies regarding the follow‐up periods, PFA was significantly associated with a 25% reduction in ATA recurrence compared to HPSD ablation (45–90 W) at 1 year (RR: 0.75 with 95% CI [0.61, 0.92], *p* < 0.01); PFA showed a trend toward an association with a lower incidence of ATA recurrence but not significant compared to HPSD ablation (45–90 W) at 6 months (RR: 0.65 with 95% CI [0.43, 1.00], *p* = 0.05) (Figure 1B). Pooled studies were homogenous at 6 months (*I* ^2^ = 0%, *p* = 0.73) and 1 year (*I* ^2^ = 17%, *p* = 0.31).

A funnel plot was utilized to assess potential publication bias. We found no significant asymmetry by inspection with no significant publication bias (Egger's *p*‐value > 0.1) (Supporting Information S1: Figure S3).

Our reconstructed time‐to‐event analysis, pooling six studies [41, 42, 44, 45, 46, 47], showed that overall freedom from ATA recurrence for the PFA group at 18 months of follow‐up was 81.9%, [78.9%, 85.0%], and for the HPSD ablation (45–90 W) group, 67.6%, [60.0%, 76.1%]. Table 4 provides detailed freedom from ATA recurrence for each 3‐month follow‐up interval. The log‐rank test showed a statistically significant difference between PFA and HPSD ablation (45–90 W) in freedom from ATA recurrence (*p* = 0.003) (Figure 1C).

**Table 4:** Probability of freedom from any atrial tachyarrhythmia recurrence for each three‐month interval of follow‐up from a reconstructed time‐to‐event model.

Study arms	6 months	9 months	12 months	15 months	18 months
PFA	91.0%, [88.9%, 93.2%]	86.2%, [83.6%, 88.9%]	81.9%, [78.9%, 85.0%]	81.9%, [78.9%, 85.0%]	81.9%, [78.9%, 85.0%]
HPSD (45–90 W) ablation.	89.5%, [87.4%, 91.6%]	81.4%, [78.8%, 84.1%]	74.7%, [71.7%, 77.8%]	72.8%, [69.0%, 76.9%]	67.6%, [60.0%, 76.1%]
HPSD (45–50 W) ablation.	91.5%, [89.3%, 93.8%]	83.3%, [80.3%, 86.4%]	75.8%, [72.4%, 79.4%]	—	—
vHPSD (70–90 W) ablation.	84.6%, [80.2%, 89.1%]	77.6%, [72.5%, 83.0%]	73.5%, [67.9%, 79.7%]	71.4%, [65.3%, 78.2%]	66.3%, [57.7%, 76.1%]

Abbreviation: HPSD: high‐power short duration; PFA: pulsed‐field ablation; vHPSD: very high‐power short duration.

Our Cox frailty regression model showed that PFA was significantly associated with a 28% lower risk of ATA recurrence compared to HPSD ablation (45–90 W) (HR: 0.72 with 95% CI [0.57, 0.91], *p* = 0.006) over 18 months of follow‐up (Figure 1C). The variance of the random effect (frailty) was 0.15, indicating some unobserved heterogeneity. Moreover, statistically significant heterogeneity was observed between studies (likelihood ratio test, *p* < 0.001) (Supporting Information S1: Table S5).

However, upon investigating the Schoenfeld residuals plot and log‐log survival curve, the proportional hazards assumption over time was visually violated, and the Grambsch‐Therneau test was statistically significant (*p* = 0.012) (Supporting Information S1: Figures S4 and S5).

**Figure 2:**
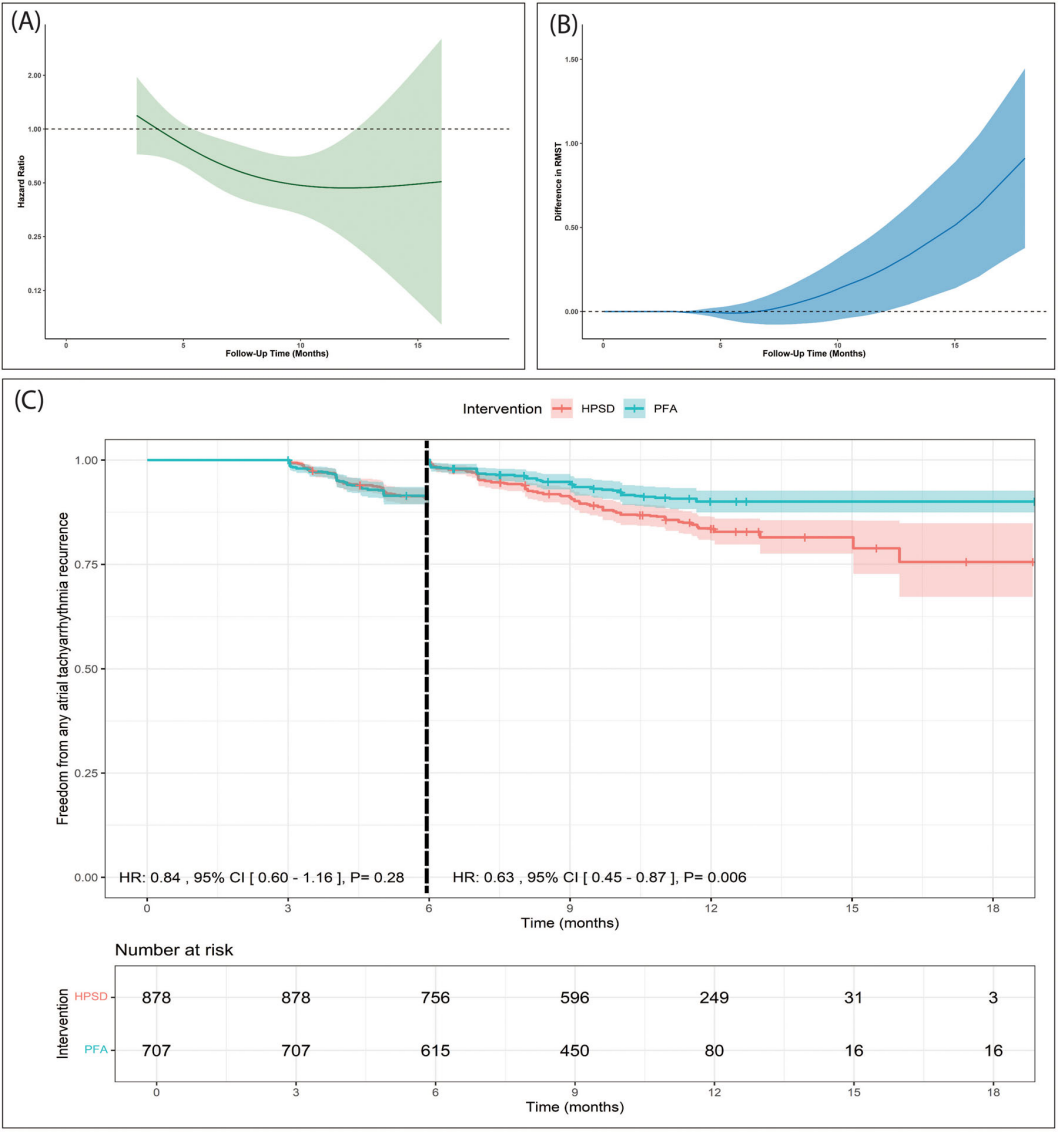
(A) Analysis of time‐varying hazard ratios for any atrial tachyarrhythmia recurrence based on flexible parametric survival models with B‐splines. (B) Restricted mean survival time over the follow‐up period. (C) Landmark analysis. HR, hazard ratio.

Figure 2A shows time‐varying HRs for ATA recurrence based on flexible parametric survival models with B‐splines, which revealed a statistically significant decrease in the risk of ATA recurrence for PFA compared to HPSD ablation (45–90 W) (HR < 1) over the follow‐up period.

The difference in RMST over the follow‐up period is presented in Figure 2B, revealing that PFA was associated with a statistically significant longer survival time from ATA recurrence of 1.035 months (95% CI, 0.444–1.626, *p* = 0.001) over the follow‐up period compared to HPSD ablation (45–90 W).

We performed a landmark analysis and designated 6 months as the landmark time point. From a 3‐month blanking period to 6 months, we found no significant difference between PFA and HPSD ablation (45–90 W) in the risk of ATA recurrence (HR: 0.84 with 95% CI [0.60, 1.16], *p* = 0.28). However, after 6 months, we found that PFA was significantly associated with a 37% decrease in the risk of ATA recurrence compared to HPSD ablation (45–90 W) (HR: 0.63 with 95% CI [0.45, 0.87], *p* = 0.006) (Figure 2C).

Jackknife analysis, where each study was excluded iteratively, showed consistent results (Supporting Information S1: Table S6 and Figure S6). The meta‐regression analysis revealed no significant modulating effect of any baseline variable on the RR between PFA and HPSD ablation (45–90 W) for ATA recurrence (Supporting Information S1: Table S7 and Figures S7–S15).

Upon conducting a sensitivity analysis and considering the first month as the blanking period, the log‐rank test revealed a statistically significant difference in freedom from ATA recurrence between PFA and HPSD ablation (45–90 W) (*p* = 0.005) (Supporting Information S1: Figure S16). Our Cox frailty regression model showed that PFA was significantly associated with a 26% lower risk of ATA recurrence compared to HPSD ablation (45–90 W) (HR: 0.74, 95% CI [0.59, 0.92], *p* = 0.008) over 18 months of follow‐up (Supporting Information S1: Figure S16).

**Figure 3:**
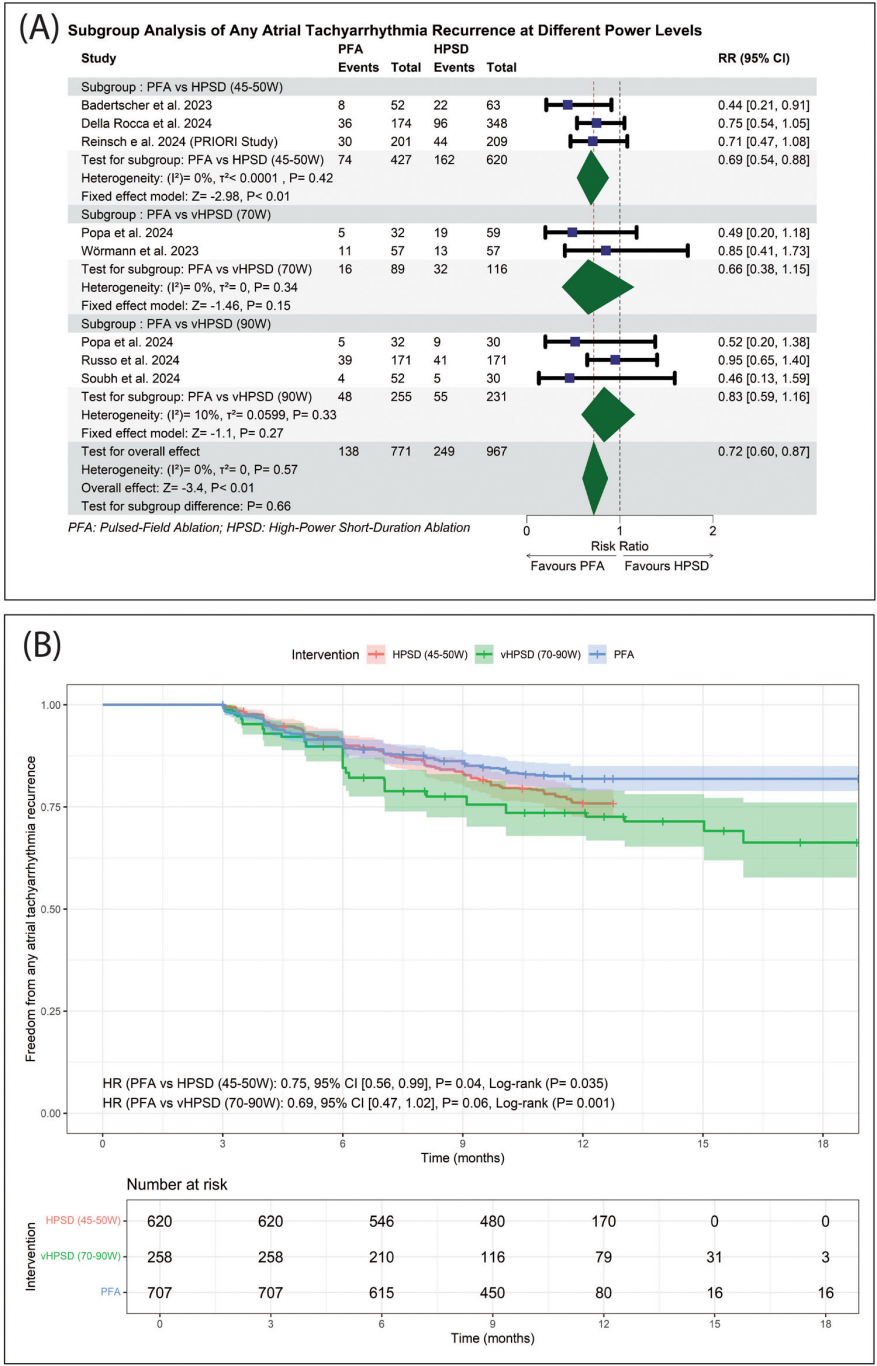
(A) Forest plot of subgroup analysis for any atrial tachyarrhythmia recurrence regarding the power levels. (B) Sub‐grouped Kaplan‐Meier curve showing the freedom from any atrial tachyarrhythmia recurrence at different power levels. HR, hazard ratio.

Further pairwise sub‐group analysis regarding the HPSD ablation (45–90 W) power levels; (HPSD ablation (45–50 W), vHPSD ablation (70 W), and vHPSD ablation (90 W)) showed that PFA was significantly associated with a 31% reduction in ATA recurrence compared to HPSD ablation (45–50 W) (RR: 0.69 with 95% CI [0.54, 0.88], *p* < 0.01). However, there was no significant difference between PFA and vHPSD ablation (70 W) (RR: 0.66 with 95% CI [0.38, 1.15], *p* = 0.15) and vHPSD ablation (90 W) (RR: 0.83 with 95% CI [0.59, 1.16], *p* = 0.27), with no significant subgroup difference (*p* = 0.66) (Figure 3A).

Our reconstructed time‐to‐event analysis, pooling six studies [41, 42, 44, 45, 46, 47], showed that overall freedom from ATA recurrence for the PFA group at 18 months of follow‐up was 81.9%, [78.9%, 85.0%], for the HPSD ablation (45–50 W) at 12 months was 75.8%, [72.4%, 79.4%], and for the vHPSD ablation (70–90 W) at 18 months was 66.3%, [57.7%, 76.1%]. Table 4 provides detailed freedom from ATA recurrence for each 3‐month follow‐up interval. However, the log‐rank test showed a statistically significant difference between PFA and HPSD ablation (45–50 W) (*p* = 0.035), as well as between PFA and vHPSD ablation (70–90 W) (*p* = 0.001); our Cox frailty regression model, after including the γ frailty term, where individual studies are modelled as a random effect, showed that PFA was significantly associated with a 25% lower risk of ATA recurrence compared to the HPSD ablation (45–50 W) (HR: 0.75 with 95% CI [0.56, 0.99], *p* = 0.04) with no significant difference between PFA and vHPSD ablation (70–90 W) (HR: 0.69 with 95% CI [0.47, 1.02], *p* = 0.06) (Figure 3B).

Upon conducting sensitivity analysis and considering the first month as the blanking period, our Cox frailty regression model, after including the γ frailty term, where individual studies are modelled as a random effect, showed that PFA showed a trend toward an association with a lower risk of ATA recurrence compared to the HPSD ablation (45–50 W) (HR: 0.77 with 95% CI [0.58, 1.00], *p* = 0.05) and a lower risk of ATA recurrence compared to vHPSD ablation (70–90 W) (HR: 0.67 with 95% CI [0.47, 0.97], *p* = 0.03) (Supporting Information S1: Figure S17).

**Figure 4:**
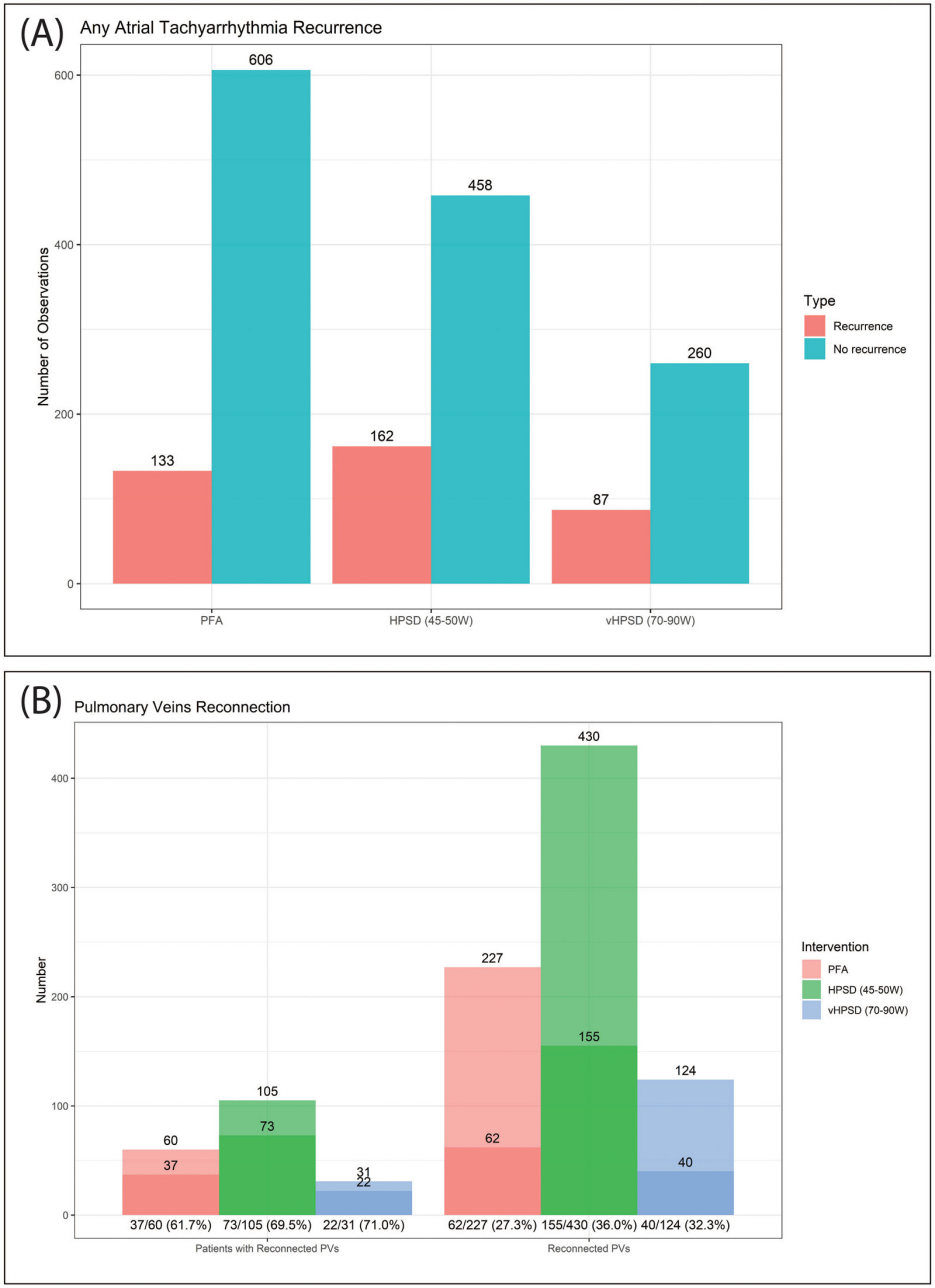
(A) Bar graph showing any atrial tachyarrhythmia recurrence rates. (B) Bar graph showing pulmonary veins reconnection status.

Our pooled analysis showed that ATA recurrence rates were (18.0%), (26.1%), and (25.1%) for PFA, HPSD ablation (45–50 W), and vHPSD ablation (70–90 W), respectively (Figure 4A).

Furthermore, among patients who underwent repeat ablation procedures, the percentage of patients with reconnected PVs was (61.7%), (69.5%), and (71.0%) for PFA, HPSD ablation (45–50 W), and vHPSD ablation (70–90 W), respectively, with no significant difference between PFA and either HPSD or vHPSD ablation. However, the percentage of reconnected PVs was (27.3%), (36.0%), and (32.3%) for PFA, HPSD ablation (45–50 W), and vHPSD ablation (70–90 W), respectively, showing a significant difference between PFA and HPSD ablation (45–50 W) (*p* = 0.03), though there was no significant difference between PFA and vHPSD ablation (70–90 W) (Figure 4B).

### 3.5 Secondary Outcomes

#### 3.5.1 Procedural Outcomes

**Figure 5:**
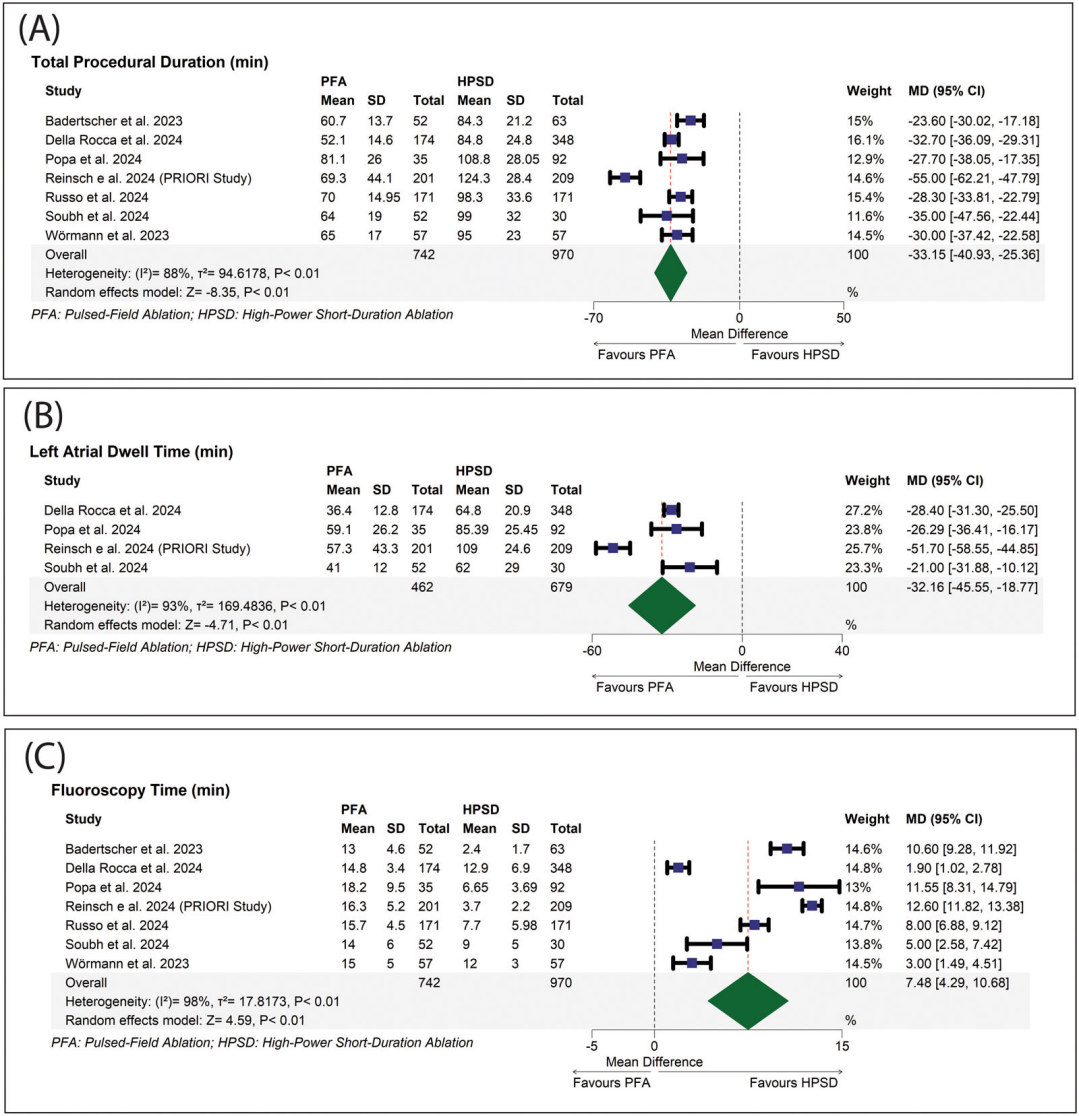
(A) Forest plot of total procedural duration. (B) Forest plot of left atrial dwell time. (C) Forest plot of fluoroscopy time.

Compared to HPSD ablation (45–90 W), PFA was significantly associated with reduced total procedural duration (MD: −33.15 with 95% CI [−40.93, −25.36], *p* < 0.01) (Figure 5A) and reduced left atrial dwell time (MD: −32.16 with 95% CI [−45.55, −18.77], *p* < 0.01) (Figure 5B). In contrast, PFA was significantly associated with increased fluoroscopy time compared to HPSD ablation (45–90 W) (MD: 7.48 with 95% CI [4.29, 10.68], *p* < 0.01) (Figure 5C).

Pooled studies were heterogeneous in total procedural duration (*I* ^2^ = 88%, *p* < 0.01), left atrial dwell time (*I* ^2^ = 93%, *p* < 0.01), and fluoroscopy time (*I* ^2^ = 98%, *p* < 0.01). Regarding total procedural time, heterogeneity was resolved after excluding Reinsch et al. (*I* ^2^ = 32%) (Supporting Information S1: Figure S18). Regarding left atrial dwell time, heterogeneity was resolved after excluding Reinsch et al. (*I* ^2^ = 0%) (Supporting Information S1: Figure S19). Regarding fluoroscopy time, sensitivity analysis did not resolve the heterogeneity (Supporting Information S1: Figure S20).

Further subgroup analysis for the different power levels showed no significant difference (*p* > 0.1) (Supporting Information S1: Figures S21–S23). Regarding total procedural duration, left atrial dwell time, and fluoroscopy time, we found no significant asymmetry by inspection, with no significant publication bias (Egger's *p* value > 0.1) (Supporting Information S1: Figures S24–S26).

#### 3.5.2 Safety Outcomes

**Figure 6:**
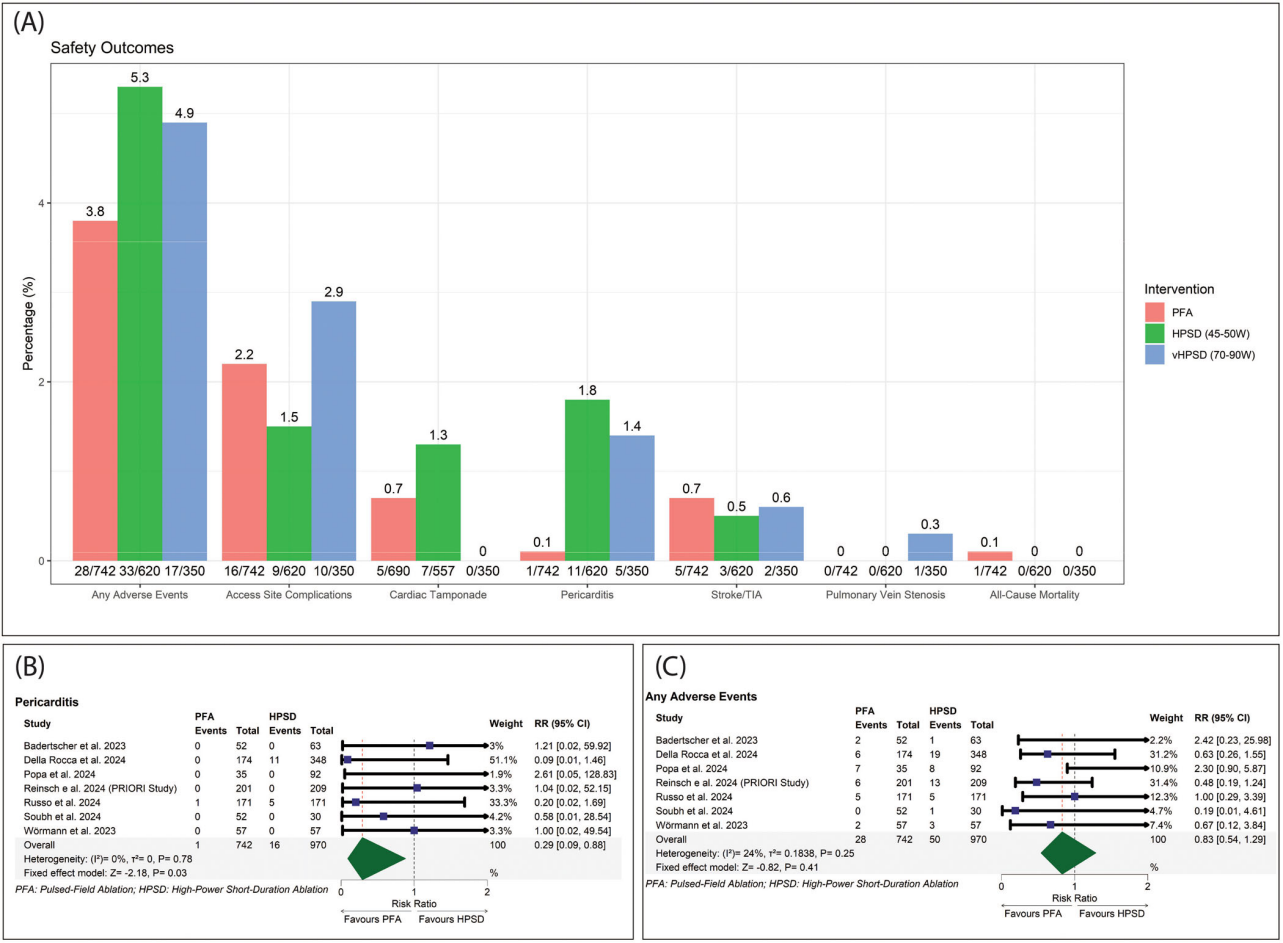
(A) Bar graph showing the safety profiles. (B) Forest plot of pericarditis. (C) Forest plot of any adverse events.

Our pooled analysis showed that any AEs rates were (3.8%), (5.3%), and (4.9%) for PFA, HPSD ablation (45–50 W), and vHPSD ablation (70–90 W), respectively, access site complications rates were (2.2%), (1.5%), and (2.9%) for PFA, HPSD ablation (45–50 W), and vHPSD ablation (70–90 W), respectively, cardiac tamponade rates were (0.7%), (1.3%), and (0%) for PFA, HPSD ablation (45–50 W), and vHPSD ablation (70–90 W), respectively, pericarditis rates were (0.1%), (1.8%), and (1.4%) for PFA, HPSD ablation (45–50 W), and vHPSD ablation (70–90 W), respectively, stroke/TIA rates were (0.7%), (0.5%), and (0.6%) for PFA, HPSD ablation (45–50 W), and vHPSD ablation (70–90 W), respectively, PV stenosis rates were (0%), (0%), and (0.3%) for PFA, HPSD ablation (45–50 W), and vHPSD ablation (70–90 W), respectively, and all‐cause mortality rates were (0.1%), (0%), and (0%) for PFA, HPSD ablation (45–50 W), and vHPSD ablation (70–90 W), respectively. However, the chi‐squared test showed no significant difference between PFA and either HPSD or vHPSD ablation across the safety profile; it showed a statistically significant difference between PFA and HPSD ablation (45–50 W) (*p* = 0.003), as well as between PFA and vHPSD ablation (70–90 W) (*p* = 0.019) in pericarditis (Figure 6A).

PFA was significantly associated with a lower incidence of pericarditis (RR: 0.29 with 95% CI [0.09, 0.88], *p* = 0.03) (Figure 6B). However, there was no significant difference between PFA and HPSD ablation (45–90 W) in any AEs (RR: 0.83 with 95% CI [0.54, 1.29], *p* = 0.41) (Figure 6C), access site complications (RR: 1.39 with 95% CI [0.75, 2.58], *p* = 0.30) (Supporting Information S1: Figure S27), cardiac tamponade (RR: 0.89 with 95% CI [0.34, 2.31], *p* = 0.81) (Supporting Information S1: Figure S28), stroke/TIA (RR: 1.25 with 95% CI [0.48, 3.28], *p* = 0.64) (Supporting Information S1: Figure S29), PV stenosis (RR: 0.91 with 95% CI [0.24, 3.51], *p* = 0.89) (Supporting Information S1: Figure S30), and all‐cause mortality (RR: 1.52 with 95% CI [0.38, 5.99], *p* = 0.55) (Supporting Information S1: Figure S31).

Pooled studies were homogenous in pericarditis (*I* ^2^ = 0%, *p* = 0.78), any AEs (*I* ^2^ = 24%, *p* = 0.25), access site complications (*I* ^2^ = 0%, *p* = 0.55), cardiac tamponade (*I* ^2^ = 0%, *p* = 0.82), stroke/TIA (*I* ^2^ = 0%, *p* = 0.82), PV stenosis (*I* ^2^ = 0%, *p* = 0.99), and all‐cause mortality (*I* ^2^ = 0%, *p* = 1.00).

Further subgroup analysis for the different power levels showed no significant difference in any AEs (*p* = 0.11) (Supporting Information S1: Figure S32). Regarding PV stenosis and pericarditis, funnel plots were utilized to assess potential publication bias. Significant asymmetry was observed upon inspection, indicating a potential publication bias (Egger's *p*‐value = 0.04) and (Egger's *p*‐value = 0.03), respectively. To address this, the trim and fill method was applied, as detailed in (Supporting Information S1: Figures S33–S36). Regarding other safety outcomes, we found no significant asymmetry by inspection, with no significant publication bias (Egger's *p*‐value > 0.1) (Supporting Information S1: Figures S37–41).

## 4 Discussion

Our meta‐analysis investigated the efficacy and safety of PFA compared to HPSD ablation (45–90 W) for AF. Pairwise analysis revealed that PFA was significantly associated with a lower incidence of ATA recurrence compared to HPSD ablation (45–90 W) at the longest follow‐up. Subgroup analysis revealed no significant difference in ATA recurrence between PFA and HPSD ablation (45–90 W) at 6 months; however, PFA was significantly associated with lower ATA recurrence at 1 year. Furthermore, PFA was significantly associated with lower ATA recurrence compared to HPSD ablation (45–50 W), with no significant difference observed between PFA and vHPSD ablation (70 W or 90 W). PFA was also significantly associated with shorter procedural duration and left atrial dwell time, though with increased fluoroscopy time compared to HPSD ablation (45–90 W), with no significant difference across power levels. Both techniques had similar safety profiles; however, PFA showed significantly lower rates of pericarditis compared to HPSD (45–50 W) and vHPSD (70–90 W). The pairwise analysis also showed that PFA was significantly associated with a lower incidence of pericarditis than HPSD ablation (45–90 W). A pooled reconstructed Kaplan–Meier analysis further demonstrated a 28% lower risk of ATA recurrence with PFA compared to HPSD (45–90 W) over an 18‐month follow‐up period.

PFA and HPSD ablation represent the latest advancements in the treatment of AF. Both technologies have shown the ability to achieve durable PVI and have produced favorable rhythm outcomes in patients with paroxysmal AF [32, 48, 49]. Since the PFA was reintroduced in 2019 [29], increasing clinical evidence has highlighted its safety and effectiveness as a novel ablation method [32].

Our analysis showed that PFA was significantly associated with a lower incidence of ATA recurrence compared to HPSD ablation (45–90 W) at the longest follow‐up. Kaplan–Meier analysis showed that PFA was significantly associated with a 28% lower risk of ATA recurrence compared to HPSD ablation (45–90 W) over 18 months of follow‐up. Subgroup analysis in both the pairwise analysis and sub‐grouped Kaplan–Meier curve across different power levels revealed that PFA was significantly associated with lower ATA recurrence compared to HPSD ablation (45–50 W), with no significant difference in ATA recurrence between PFA and vHPSD ablation (70–90 W).

The MANIFEST‐PF registry demonstrated an arrhythmia‐free survival rate of 78% in a diverse cohort of patients with paroxysmal and persistent AF treated with PFA [32]. Similarly, the EU‐PORIA multicenter registry reported 80% arrhythmia‐free survival rates in patients with paroxysmal AF treated with PFA [31].

AF radiofrequency ablation creates lesions through two phases. In the first resistive phase, the catheter delivers heat directly to the tissue, causing immediate, irreversible injury to the superficial layer when local temperatures exceed 50°C. In the second phase, called the conductive phase, heat penetrates deeper into the tissues passively, causing reversible injury at lower temperatures (below 50°C) after the resistive phase [50].

Shortening the total procedure duration helps reduce the conductive phase, while increasing power enhances the resistive phase [51, 52]. This approach in HPSD ablation typically results in wider and shallower lesions, which may minimize conduction gaps during PV encirclement, leading to better encirclement and 100% lesion continuity [21, 52, 53]. Consequently, it creates sufficiently transmural lesions, making them more durable and safer [52].

It has been shown that a greater drop in local impedance is associated with a deeper and more durable lesion [54]. Otsuka et al. demonstrated that vHPSD ablation (90 W) results in greater impedance drops than HPSD ablation (50 W) [55]. Thus, HPSD ablation (45–50 W) may not achieve the same lesion depth as vHPSD ablation (70–90 W), potentially leading to less durable lesions. This could explain why PFA showed a significant difference compared to HPSD ablation (45–50 W) but could not reach a statistically significant difference with vHPSD ablation (70–90 W).

PV reconnection is the primary cause of AF recurrence. In our study, the percentages of patients with reconnected PVs were (61.7%), (69.5%), and (71.0%) for PFA, HPSD ablation (45–50 W), and vHPSD ablation (70–90 W), respectively, with no significant difference between PFA and either HPSD or vHPSD ablation. However, the percentage of reconnected PVs was (27.3%), (36.0%), and (32.3%) for PFA, HPSD ablation (45–50 W), and vHPSD ablation (70–90 W), respectively, showing a significant difference between PFA and HPSD ablation (45–50 W) (*p* = 0.03), though there was no significant difference between PFA and vHPSD ablation (70–90 W). This could also elucidate the reason behind the statistically significant difference observed between PFA and HPSD ablation (45–50 W) but not between PFA and vHPSD ablation (70–90 W). Vagal denervation has also been shown to occur less frequently with PFA compared to radiofrequency ablation, which may play a role in improved arrhythmia‐free survival in PFA [56].

Our study showed that PFA was significantly associated with shorter procedural and left atrial dwell time, with no significant difference across the power levels. Our study was in line with the previous MANIFEST trial [32], which also showed a short procedure duration. These findings suggest that while both techniques are rapid procedures, the single‐shot design of the PFA device, along with its easy maneuverability and PV cannulation in expert hands [46], may be crucial factors contributing to its superior speed.

Our study found that PFA was significantly associated with longer fluoroscopy times, with no significant difference across various power levels. This is consistent with Kottmaier et al. [21], who reported shorter fluoroscopy times with HPSD ablation. The reduced fluoroscopy use in the HPSD ablation may be attributed to the extensive experience of the operators [46], as operator expertise has been shown to decrease fluoroscopic exposure during PFA procedures [57]. Additionally, the lack of variability in total fluoroscopy dosages between PFA and HPSD ablation in Wörmann et al. could be explained by their use of an enhanced irrigated tip catheter without contact force, combined with the excessive use of angiography in the HPSD ablation arm, which likely contributed to higher fluoroscopic exposure in the HPSD ablation arm [44].

These findings could also be due to the use of electroanatomical mapping (EAM) rather than the ablation strategy during the radiofrequency procedures. The reduced procedure time for PFA, compared to HPSD ablation, may be influenced by the EAM time utilized in the HPSD groups across the included studies [42, 43, 44, 45, 46, 47]; however, Soubh et al. [45] showed that although EAM was used in all PFA procedures, the overall procedure duration remained significantly shorter in PFA than for vHPSD ablation.

Furthermore, fluoroscopy times are expected to decrease with the use of EAM [46]. Early EAM‐based PFA systems have already demonstrated improved fluoroscopy times [58, 59]. As the Farapulse system becomes better integrated with EAM technology, the need for fluoroscopy in PFA is expected to decrease, which should further reduce fluoroscopy times [42].

Our analysis revealed that the safety profiles of PFA and HPSD ablation (45–90 W) were comparable, with no significant differences observed across the different power levels. However, our pair‐wise meta‐analysis showed that PFA was significantly associated with a lower incidence of pericarditis (*p* = 0.03). Additionally, the chi‐squared test showed a significant difference between PFA and HPSD (45–50 W) ablation (*p* = 0.003), as well as vHPSD (70–90 W) ablation (*p* = 0.019) in pericarditis rates. The rate of AEs was low in all groups. Radiofrequency ablation for AF carries a risk of pericarditis, which affects approximately 1% to 10% of patients undergoing the procedure [60, 61].

However, the higher rates of access site complications in the PFA group may be attributed to the larger diameter of the Faradrive sheath and the need for sheath exchange after transseptal puncture [47].

Although our analysis did not show significant differences in safety between PFA and HPSD ablation (45–90 W), several studies have reported that PFA is associated with a higher risk of cardiac tamponade than traditional thermal ablation [28, 62]. These tamponades were often associated with extra‐stiff, straight‐tip guidewires. When a J‐tip guidewire was used instead, and the straight‐tip guidewire was discontinued, the occurrence of cardiac tamponade ceased, as noted in the findings by Reinsch et al. [46].

### 4.1 Strengths and Limitations

To the best of our knowledge, this is the first meta‐analysis to thoroughly evaluate the latest evidence on the efficacy and safety of PFA compared to HPSD ablation (45–90 W), incorporating reconstructed Kaplan‐Meier analyses and further subgrouping outcomes based on different power levels. However, our meta‐analysis has several limitations: (1) The inclusion of only observational studies introduces a potential selection bias. (2) Only two of the seven included studies, Russo et al. and Della Rocca et al., employed PSM to account for confounding variables between the PFA and HPSD ablation groups [42, 47]. (3) Significant heterogeneity was observed in some outcomes of the pairwise analysis, likely due to variations in study protocols, the use of mapping systems, follow‐up periods, and differences in blanking periods across the included studies, which warrant cautious interpretation of the results. (4) Due to insufficient data, subgroup analysis based on paroxysmal versus persistent AF was not feasible. (5) The reliance on Holter ECG recordings rather than implantable loop recorders may have led to missed episodes of asymptomatic AF recurrence. (6) All the included studies focused on ATA recurrence as the primary outcome, neglecting the AF burden. This oversight restricts understanding of the total arrhythmic burden and the severity of symptoms. (7) Two of the included studies [41, 45] did not report data on antiarrhythmic drugs (AADs) use during follow‐up. Given that AADs use can affect ATA recurrence and other outcomes, this lack of information may have influenced the efficacy results.

### 4.2 Implications for Future Research

As a single‐shot device, PFA demonstrates exceptionally fast procedural durations but is associated with higher fluoroscopy time. Despite the shorter procedural durations, the increased fluoroscopy time suggests room for improvement. While our findings are promising, more extensive, prospective, randomized clinical trials with longer follow‐up periods are needed to validate these results further and comprehensively compare these techniques, particularly in terms of long‐term outcomes and potential complications. Future studies should consider incorporating EAM into their protocols to potentially reduce fluoroscopy time and assess any impact on procedural duration. Additionally, increased global training and experience for operators could further optimize outcomes. Moreover, implantable loop recorders in future trials could enhance arrhythmia monitoring and provide a better understanding of the differences in AF control. Furthermore, assessing AF burden rather than just reporting ATA recurrence as a binary outcome could provide a better understanding of symptom severity, as it evaluates AF as a quantitative entity.

## 5 Conclusion

PFA and HPSD ablation (45–90 W) are promising techniques for AF ablation, demonstrating high efficacy and safety. PFA had shorter procedural durations compared to HPSD ablation (45–90 W), which could offer added clinical value. However, it resulted in longer fluoroscopy time. At 6 months, both techniques showed similar ATA‐free survival rates. However, PFA was significantly associated with lower ATA recurrence at 1 year. Specifically, PFA was significantly associated with lower ATA recurrence compared to HPSD ablation (45–50 W), though not when compared to vHPSD ablation (70–90 W). The overall complication rates between the two techniques were comparable. However, PFA was significantly associated with lower rates of pericarditis compared to HPSD ablation.

## Author Contributions

A.M.A. led and administered the project, conceived the idea, designed the research workflow, and searched the databases. A.M.A., S.A.B., and M.T., screened the retrieved records, extracted relevant data, and assessed the quality of evidence. A.M.A. performed the analysis. A.M.A. wrote the final manuscript. All authors have revised the final draft. All authors have read and agreed to the final version of the manuscript.

## Ethics Statement

The authors have nothing to report.

## Consent

The authors have nothing to report.

## Conflicts of Interest

Dr. Kaplan is on an advisory board for Medtronic and speaker's bureau/honoraria for Medtronic and Boston Scientific. Dr. Lakkireddy: Speaker and advisor to Abbott, Atricure, Boston sci, J&J, and Medtronic. Dr. Di Biase is a consultant for Stereotaxis, Biosense Webster, Boston Scientific, Abbott Medical, and I‐Rhythm and has received speaker honoraria/travel from Medtronic, Biotronik, Zol, Siemens, and Haemonetics. Dr. Natale: Consultant for Abbott, Biosense Webster, Biotronik, Boston Scientific, Field Medical, iRhythm, Hemonetics, Medtronic, and Pulse Bioscience. While the other authors declare no conflict of interest.

## Supporting information

Supplementary file.

## Data Availability

All data are available within the manuscript and can be obtained from the corresponding author upon a reasonable request.
