# Supplementary material for: Efficacy and Safety of Pulsed‐Field Versus High‐Power Short‐Duration Ablation for Atrial Fibrillation: A Systematic Review and Meta‐Analysis With Reconstructed Time‐to‐Event Data
Source: J Cardiovasc Electrophysiol. 2025 May 28;36(8):1762–84. doi: 10.1111/jce.16728 (PMC12337618; doi:10.1111/jce.16728)
Supplement: Supplementary file 1 — Supplementary file. [file JCE-36-1762-s001.docx]

**Title.**

**Efficacy and Safety of Pulsed-Field versus High-Power Short-Duration Ablation for Atrial Fibrillation: A Systematic Review and Meta-Analysis with Reconstructed Time-to-Event Data.**

**Running Title.**

PFA versus HPSD ablation for atrial fibrillation.

**Authors.**

Ahmed Mazen Amin^1^, Mustafa Turkmani^2,3^, Saman Al Barznji^2,3^, Sanghamitra Mohanty^4^,

Rachel M. Kaplan^5^, Jeffrey Winterfield^5^, Dhanunjaya Lakkireddy^6^, Pasquale Santangeli^7^, Luigi Di Biase^8^, Andrea Natale^4,9^.

**Affiliations.**

1. Faculty of Medicine, Mansoura University, Mansoura, Egypt.
2. Department of Internal Medicine, Michigan State University, Faculty of Medicine, East Lansing, Michigan, USA.
3. Department of Internal Medicine, McLaren Health Care, Oakland, Michigan, USA.
4. Texas Cardiac Arrhythmia Institute, St David's Medical Center, Austin, Texas, USA.
5. Section of Cardiac Electrophysiology, Division of Cardiology, Medical University of South Carolina (MUSC), Charleston, SC, USA.
6. Kansas City Heart Rhythm Institute, Overland Park, Kansas, USA.
7. Section of Cardiac Pacing and Electrophysiology, Heart and Vascular Institute, Cleveland Clinic, Cleveland, OH, USA.
8. Department of Electrophysiology, Albert Einstein College of Medicine at Montefiore Hospital, New York, New York, USA.
9. Department of Biomedicine and Prevention, Division of Cardiology, University of Tor Vergata, Rome, Italy.

**Contents:**

**Figures.**Figure S1: PRISMA flow chart of the screening process.

Figure S2: Quality assessment of the risk of bias in the included trials (ROBINS-I tool) in the pair-wise meta-analysis model. The upper panel presents a schematic representation of risks (low = green, unclear = yellow, and high = red) for specific types of biases of each study in the review. The lower panel presents risks (low = green, unclear = yellow, and high = red) for the subtypes of biases of the combination of studies included in this review.

Figure S3: Funnel plot of any atrial tachyarrhythmia recurrence.

Figure S4: Grambsch-Therneau test and diagnostic plots based on Schoenfeld residuals.

Figure S5: log-log survival curve.

Figure S6: Results from Jackknife sensitivity analysis.

Figure S7: Bubble plot of meta-regression analysis of any atrial tachyarrhythmia recurrence based on age.

Figure S8: Bubble plot of meta-regression analysis of any atrial tachyarrhythmia recurrence based on male percentage.

Figure S9: Bubble plot of meta-regression analysis of any atrial tachyarrhythmia recurrence based on body mass index.

Figure S10: Bubble plot of meta-regression analysis of any atrial tachyarrhythmia recurrence based on left ventricular ejection fraction.

Figure S11: Bubble plot of meta-regression analysis of any atrial tachyarrhythmia recurrence based on patients with hypertension.

Figure S12: Bubble plot of meta-regression analysis of any atrial tachyarrhythmia recurrence based on patients with diabetes mellitus.

Figure S13: Bubble plot of meta-regression analysis of any atrial tachyarrhythmia recurrence based on patients with coronary artery disease.

Figure S14: Bubble plot of meta-regression analysis of any atrial tachyarrhythmia recurrence based on patients with previous stroke/TIA.

Figure S15: Bubble plot of meta-regression analysis of any atrial tachyarrhythmia recurrence based on left atrium diameter (LAD).

Figure S16: Pooled Kaplan-Meier curve showing the freedom from any atrial tachyarrhythmia recurrence (after considering the first month only as the blanking period).

Figure S17: Sub-grouped Kaplan-Meier curve showing the freedom from any atrial tachyarrhythmia recurrence at different power levels (after considering the first month only as the blanking period).

Figure S18: Sensitivity analysis of total procedural duration.

Figure S19: Sensitivity analysis of left atrial dwell time.

Figure S20: Sensitivity analysis of fluoroscopy time.

Figure S21: Subgroup analysis of total procedural duration at different power levels.

Figure S22: Subgroup analysis of left atrial dwell time at different power levels.

Figure S23: Subgroup analysis of fluoroscopy time at different power levels.

Figure S24: Funnel plot of total procedure time.

Figure S25: Funnel plot of left atrial dwell time.

Figure S26: Funnel plot of fluoroscopy time.

Figure S27: Forest plot of access site complications.

Figure S28: Forest plot of cardiac tamponade.

Figure S29: Forest plot of stroke/TIA.

Figure S30: Forest plot of pulmonary vein stenosis.

Figure S31: Forest plot of all-cause mortality.

Figure S32: Subgroup analysis of any adverse events at different power levels.

Figure S33: Funnel plot of Pulmonary vein stenosis.

Figure S34: Trim and fill plot of Pulmonary vein stenosis.

Figure S35: Funnel plot of pericarditis.

Figure S36: Trim and fill plot of Pericarditis.

Figure S37: Funnel plot of any adverse events.

Figure S38: Funnel plot of access site complications.

Figure S39: Funnel plot of cardiac tamponade.

Figure S40: Funnel plot of stroke.

Figure S41: Funnel plot of all-cause mortality.

**Tables.**Table S1: Search strategy.

Table S2: PFA Procedure details across the studies.

Table S3: HPSD ablation procedure details across the studies.

Table S4: Propensity Score Matching Details used in Della Rocca et al. and Russo et al.

Table S5: between-study heterogeneity assessment by frailty model.

Table S6: Results from Jackknife sensitivity analysis.

Table S7: Results from meta-regression analysis.


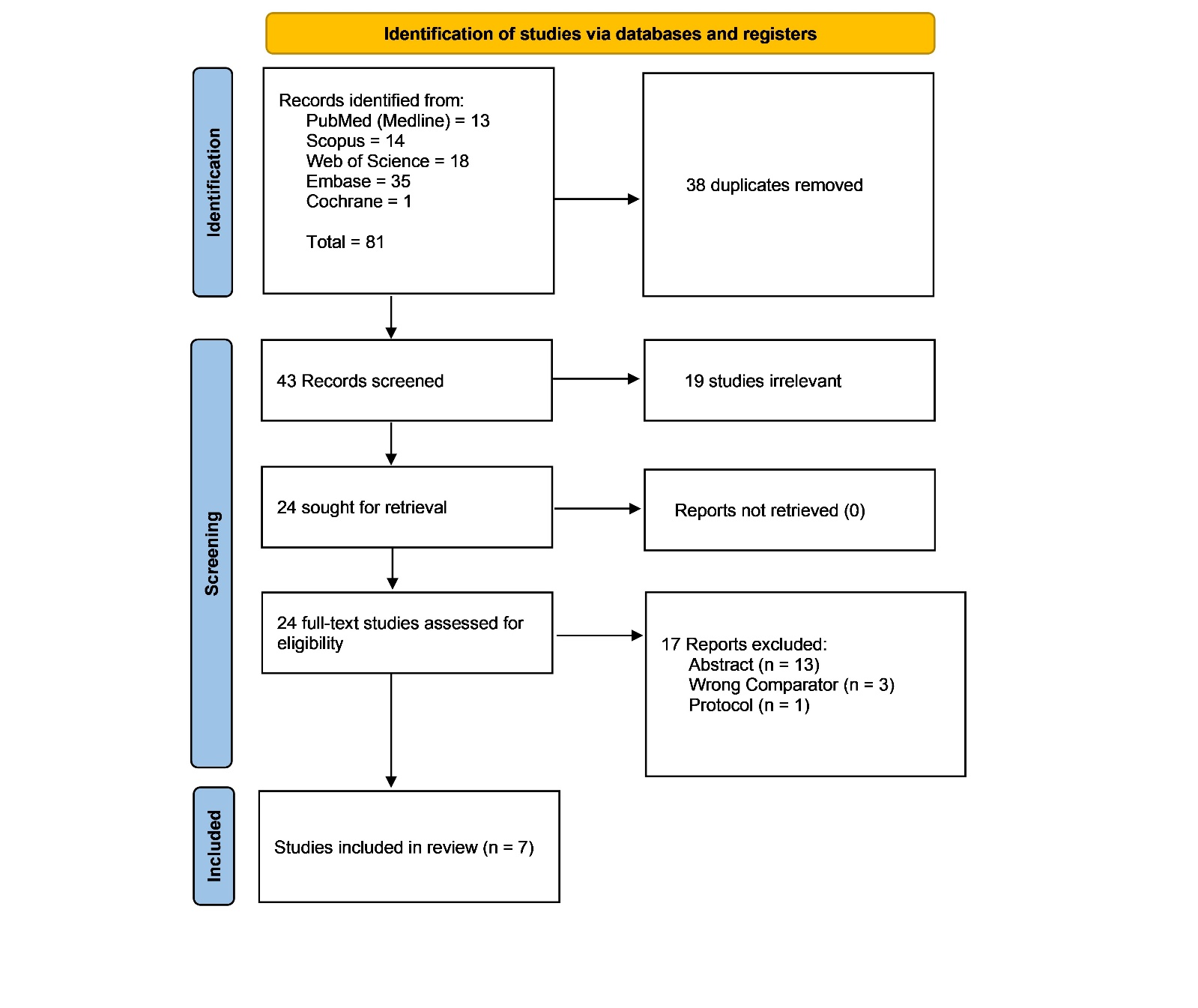
***Figure S1: PRISMA flow chart of the screening process.***


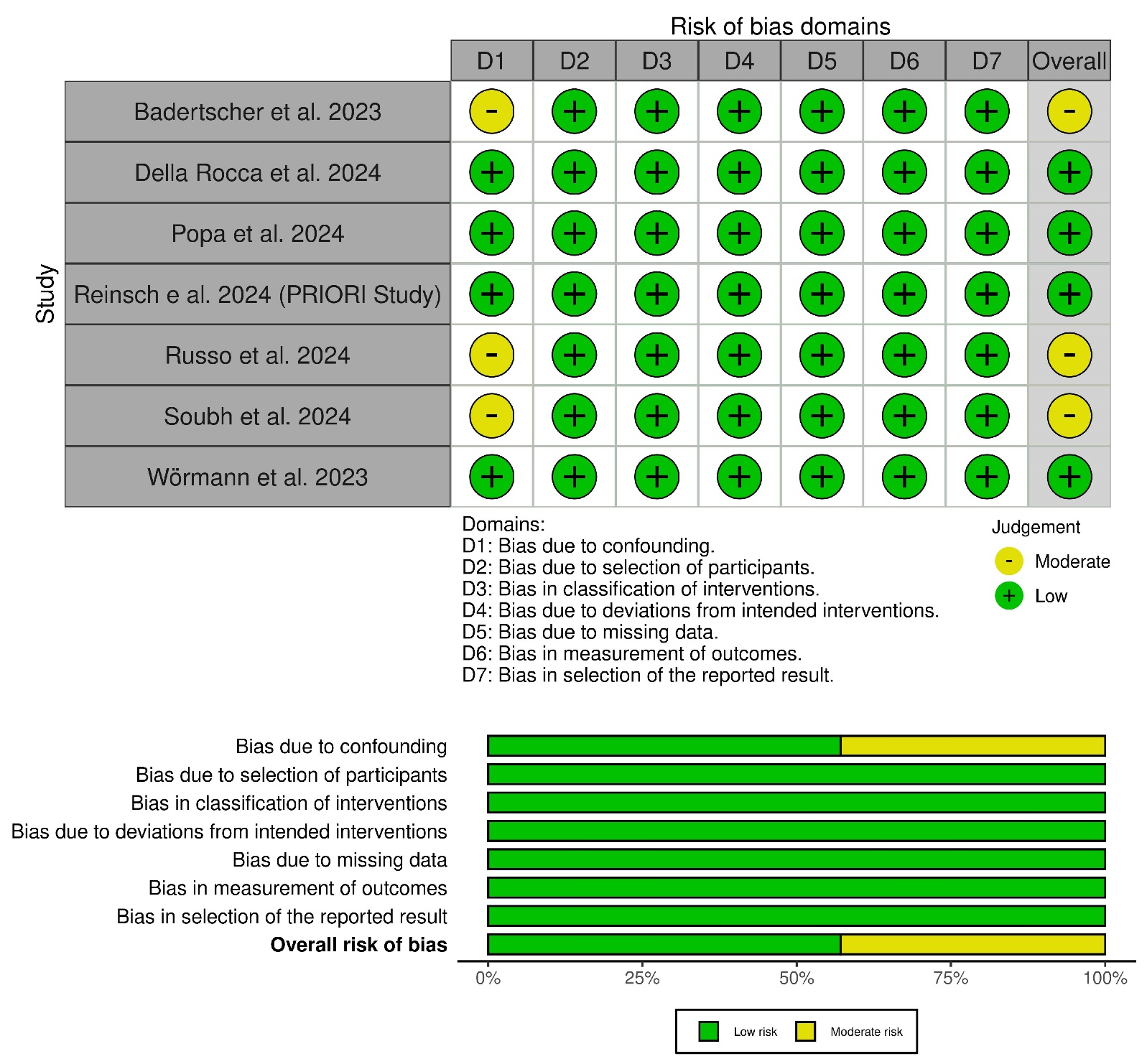


***Figure S2: Quality assessment of the risk of bias in the included trials (ROBINS-I tool) in the pair-wise meta-analysis model. The upper panel presents a schematic representation of risks (low = green, unclear = yellow, and high = red) for specific types of biases of each study in the review. The lower panel presents risks (low = green, unclear = yellow, and high = red) for the subtypes of biases of the combination of studies included in this review.***

**
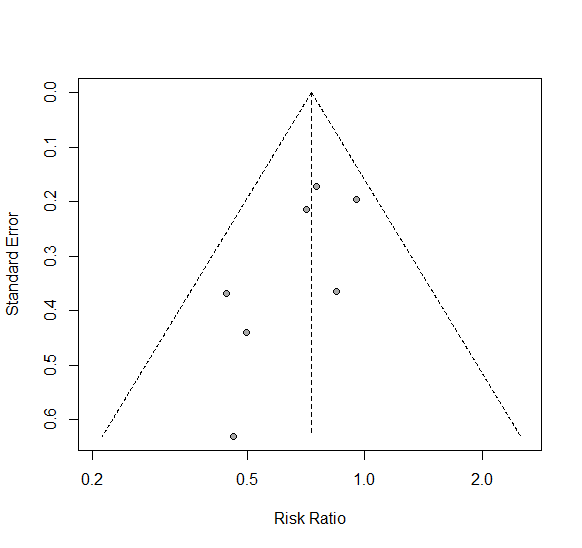
**

**Figure S3: Funnel plot of any atrial tachyarrhythmia recurrence.**


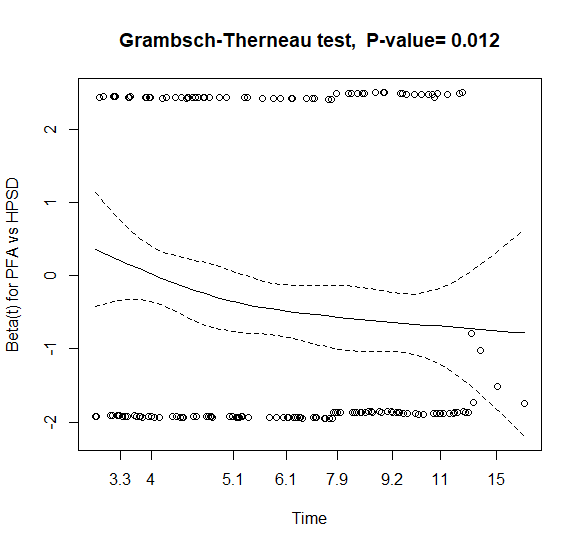


**Figure S4: Grambsch-Therneau test and diagnostic plots based on Schoenfeld residuals.**


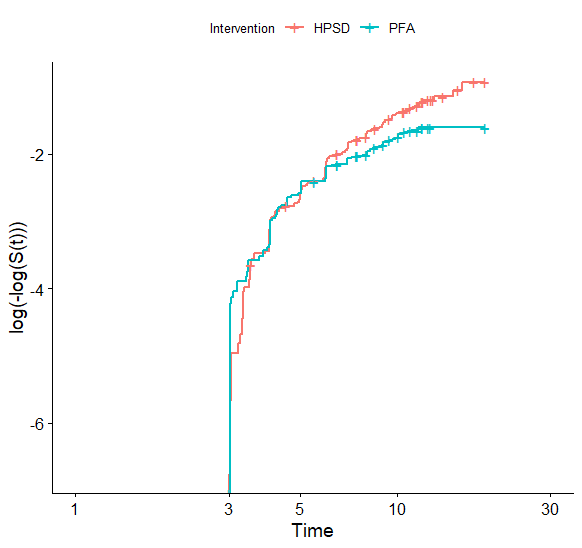


**Figure S5: log-log survival curve.**


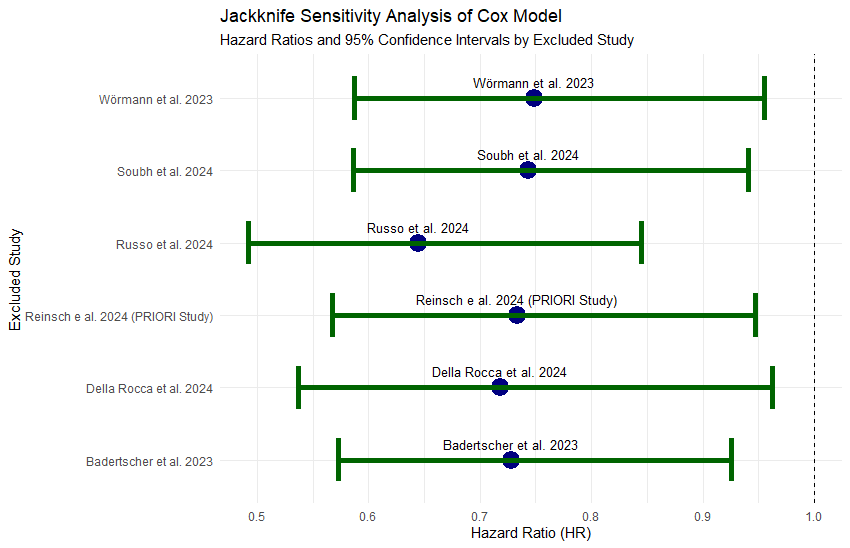


**Figure S6: Results from Jackknife sensitivity analysis.**


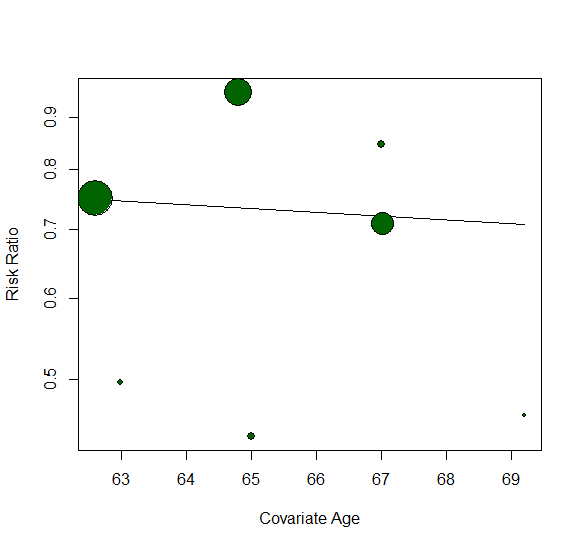


**Figure S7: Bubble plot of meta-regression analysis of any atrial tachyarrhythmia recurrence based on age.**


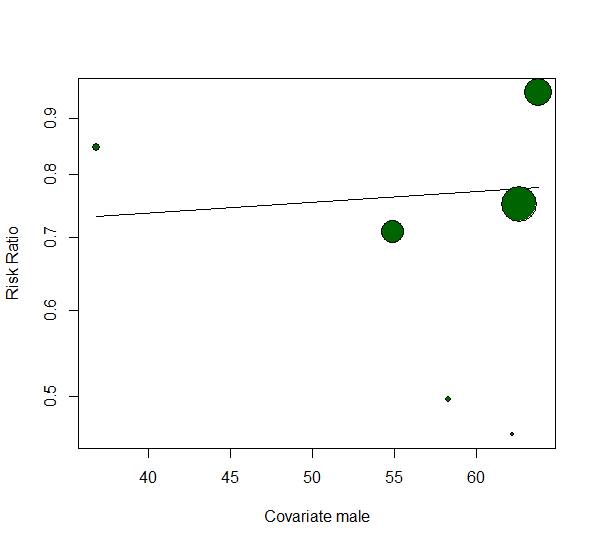


**Figure S8: Bubble plot of meta-regression analysis of any atrial tachyarrhythmia recurrence based on male percentage.**

**
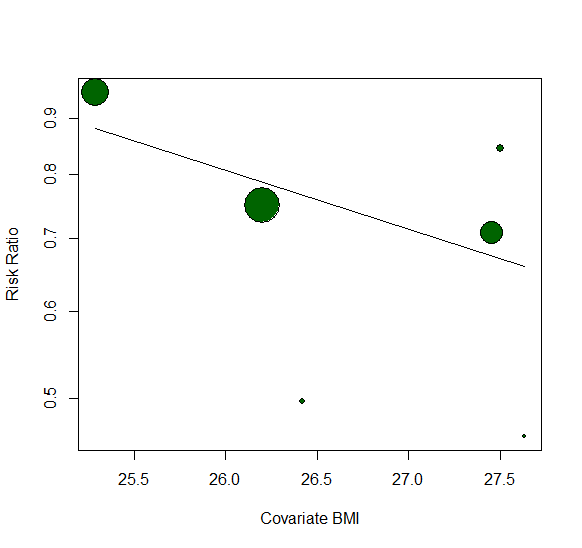
**

**Figure S9: Bubble plot of meta-regression analysis of any atrial tachyarrhythmia recurrence based on body mass index.**


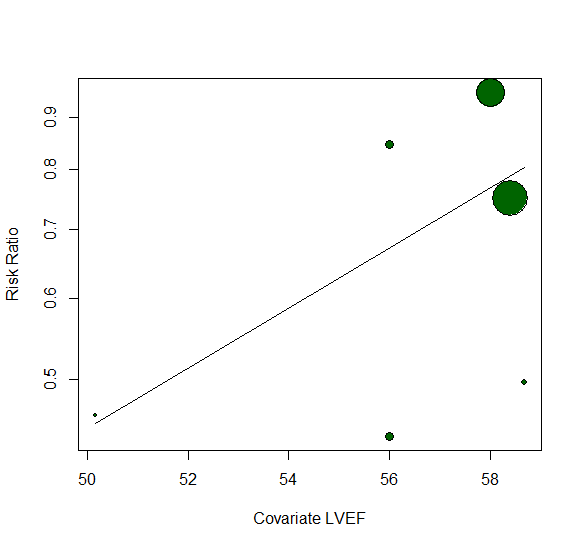


**Figure S10: Bubble plot of meta-regression analysis of any atrial tachyarrhythmia recurrence based on left ventricular ejection fraction.**

**
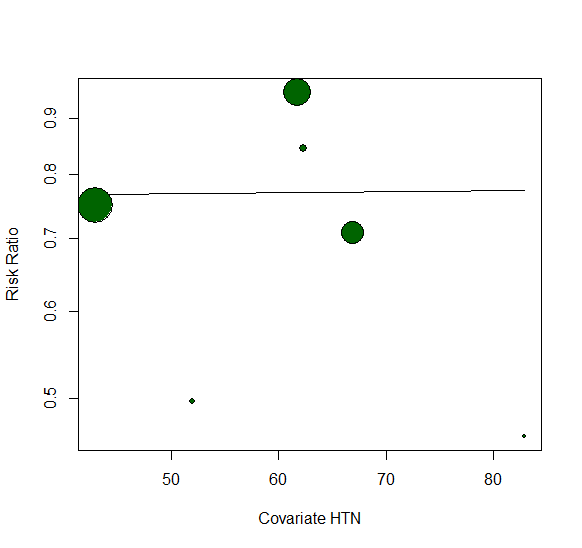
**

**Figure S11: Bubble plot of meta-regression analysis of any atrial tachyarrhythmia recurrence based on patients with hypertension.**


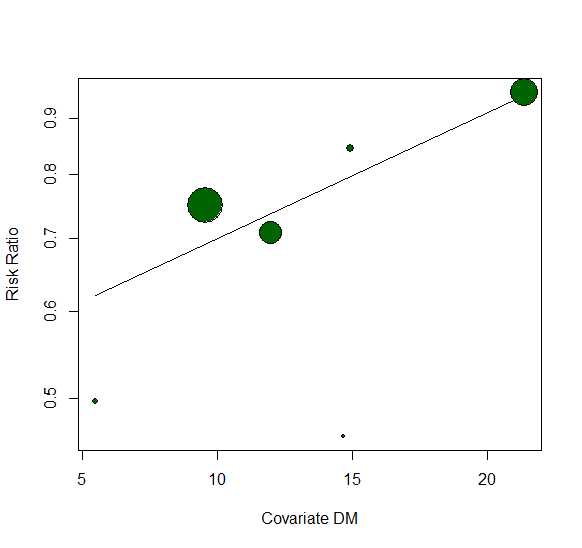


**Figure S12: Bubble plot of meta-regression analysis of any atrial tachyarrhythmia recurrence based on patients with diabetes mellitus.**


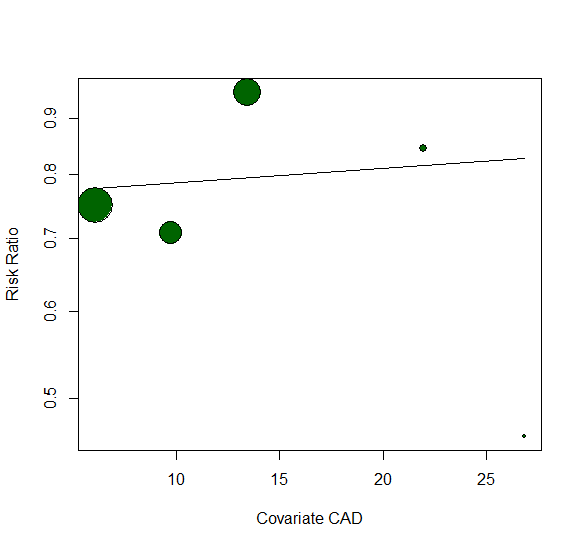


**Figure S13: Bubble plot of meta-regression analysis of any atrial tachyarrhythmia recurrence based on patients with coronary artery disease.**


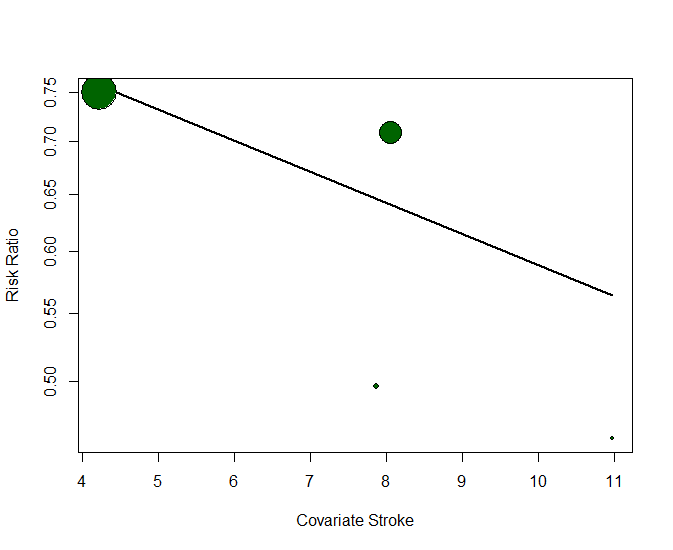


**Figure S14: Bubble plot of meta-regression analysis of any atrial tachyarrhythmia recurrence based on patients with previous stroke/TIA.**


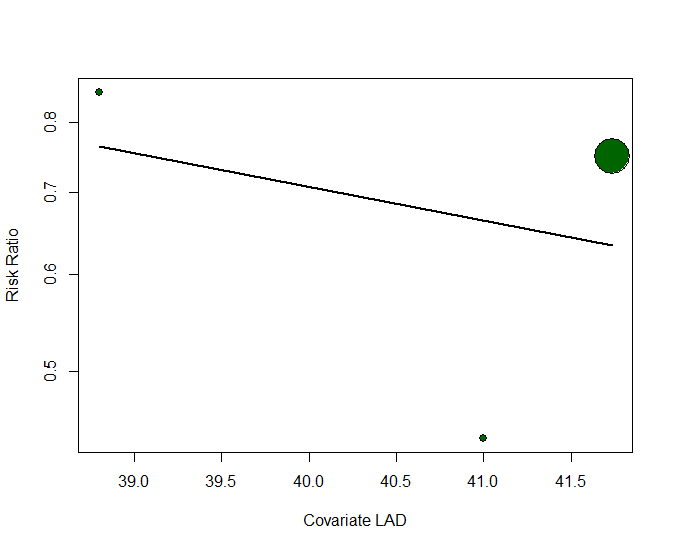


**Figure S15: Bubble plot of meta-regression analysis of any atrial tachyarrhythmia recurrence based on left atrium diameter (LAD).**


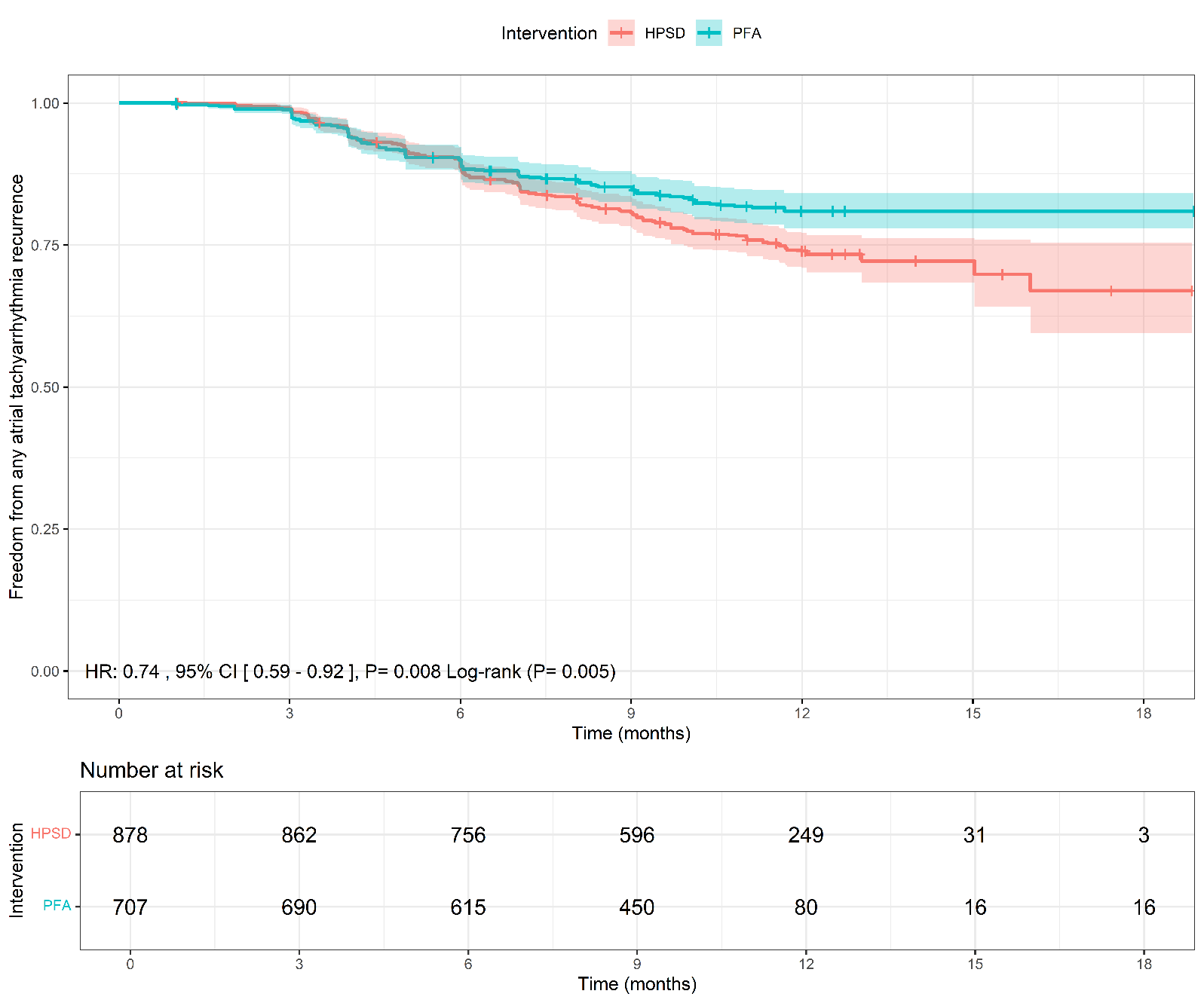


***Figure S16: Pooled Kaplan-Meier curve showing the freedom from any atrial tachyarrhythmia recurrence (after considering the first month only as the blanking period).***

***
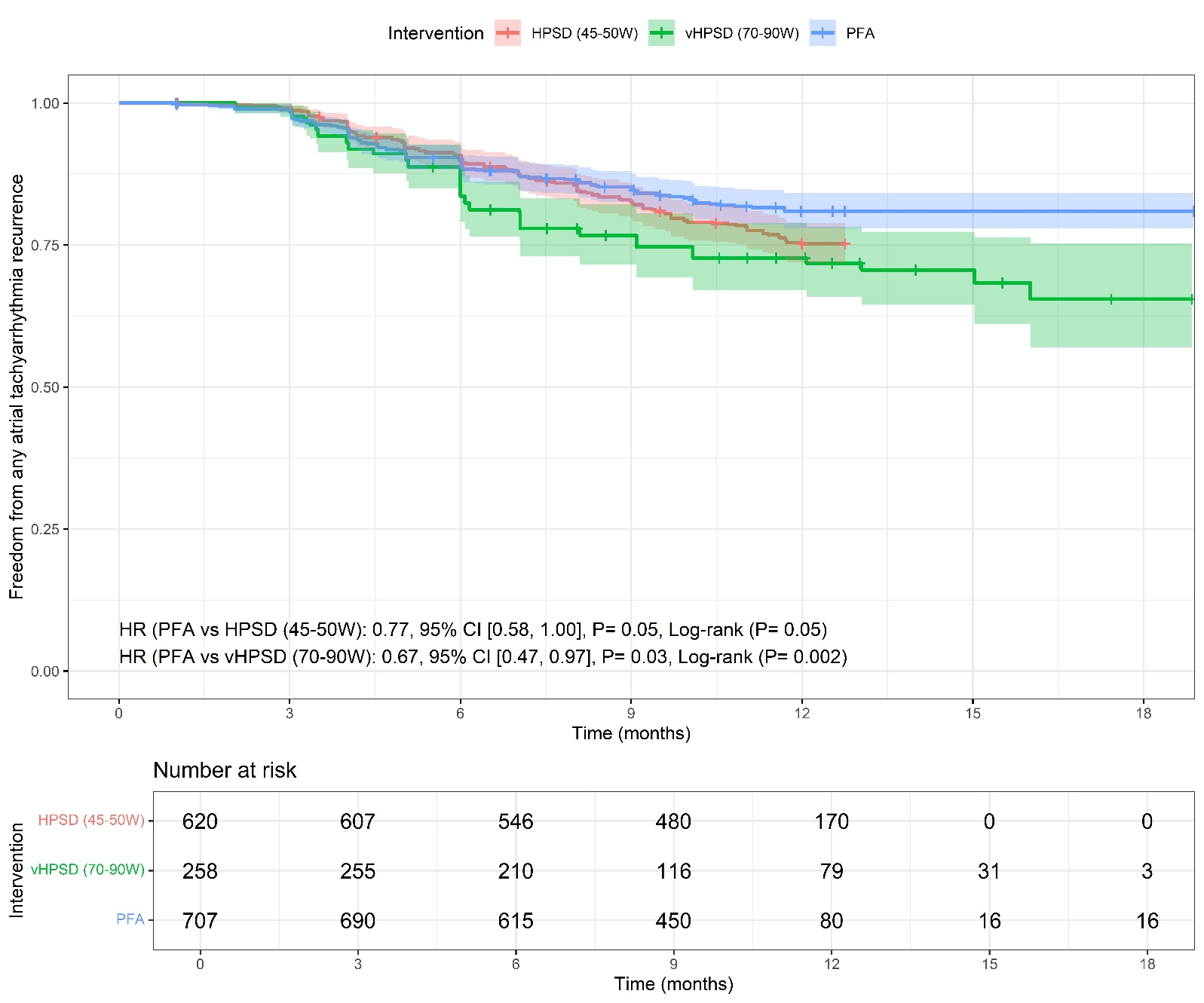
***

***Figure S17: Sub-grouped Kaplan-Meier curve showing the freedom from any atrial tachyarrhythmia recurrence at different power levels (after considering the first month only as the blanking period).***


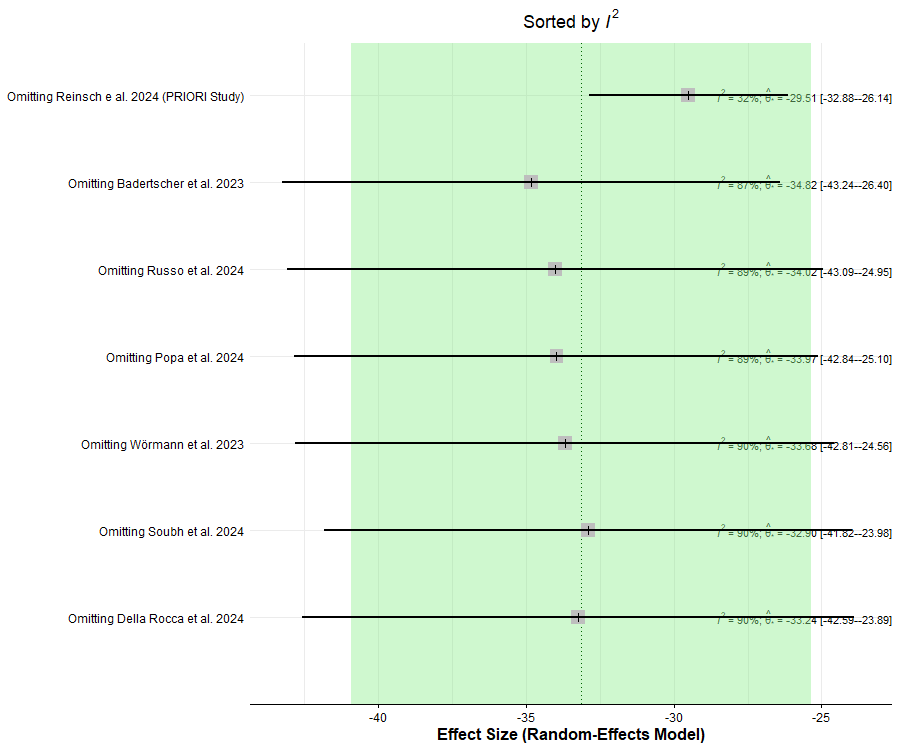


**Figure S18: Sensitivity analysis of total procedural duration.**


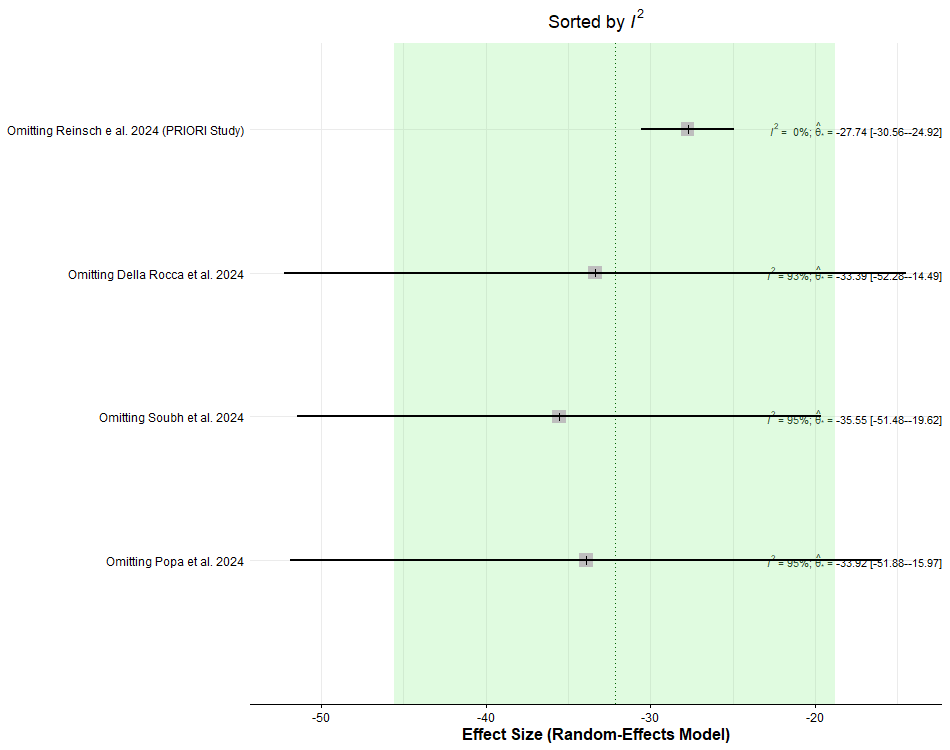


**Figure S19: Sensitivity analysis of left atrial dwell time.**


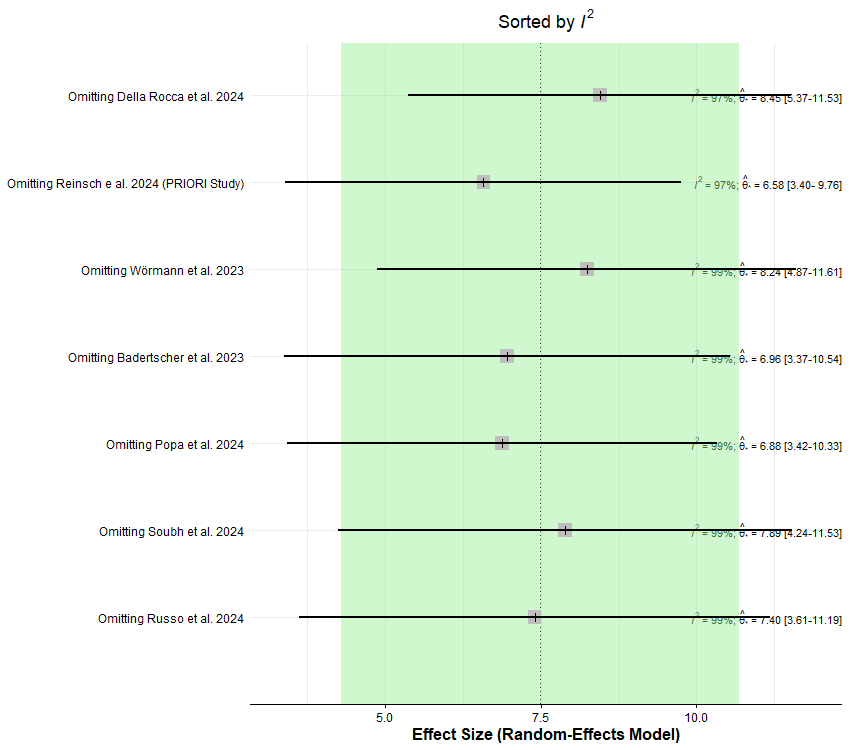


**Figure S20: Sensitivity analysis of fluoroscopy time.**





**Figure S21: Subgroup analysis of total procedural duration at different power levels.**





**Figure S22: Subgroup analysis of left atrial dwell time at different power levels.**





**Figure S23: Subgroup analysis of fluoroscopy time at different power levels.**


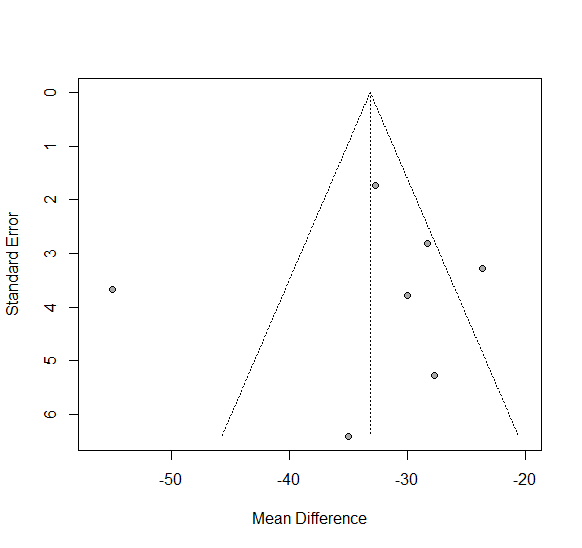


***Figure S24: Funnel plot of total procedure time.***


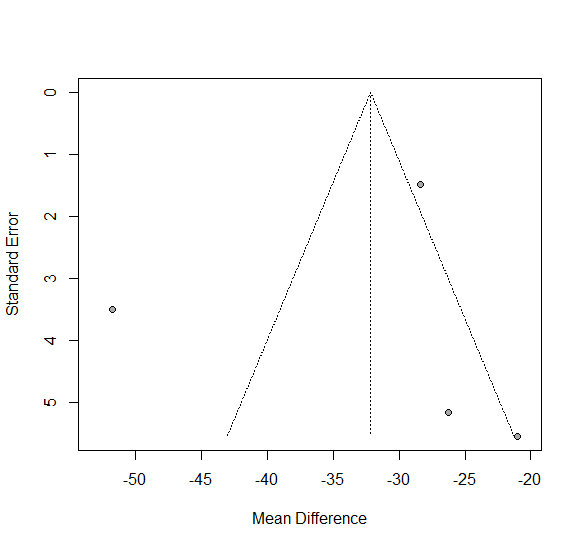


***Figure S25: Funnel plot of left atrial dwell time.***


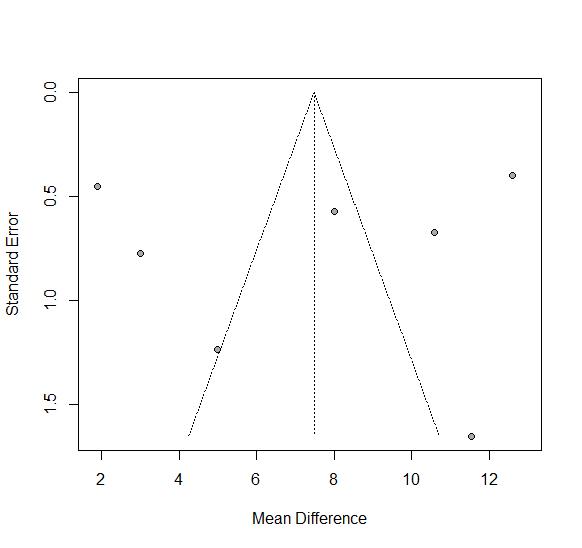


***Figure S26: Funnel plot of fluoroscopy time.***


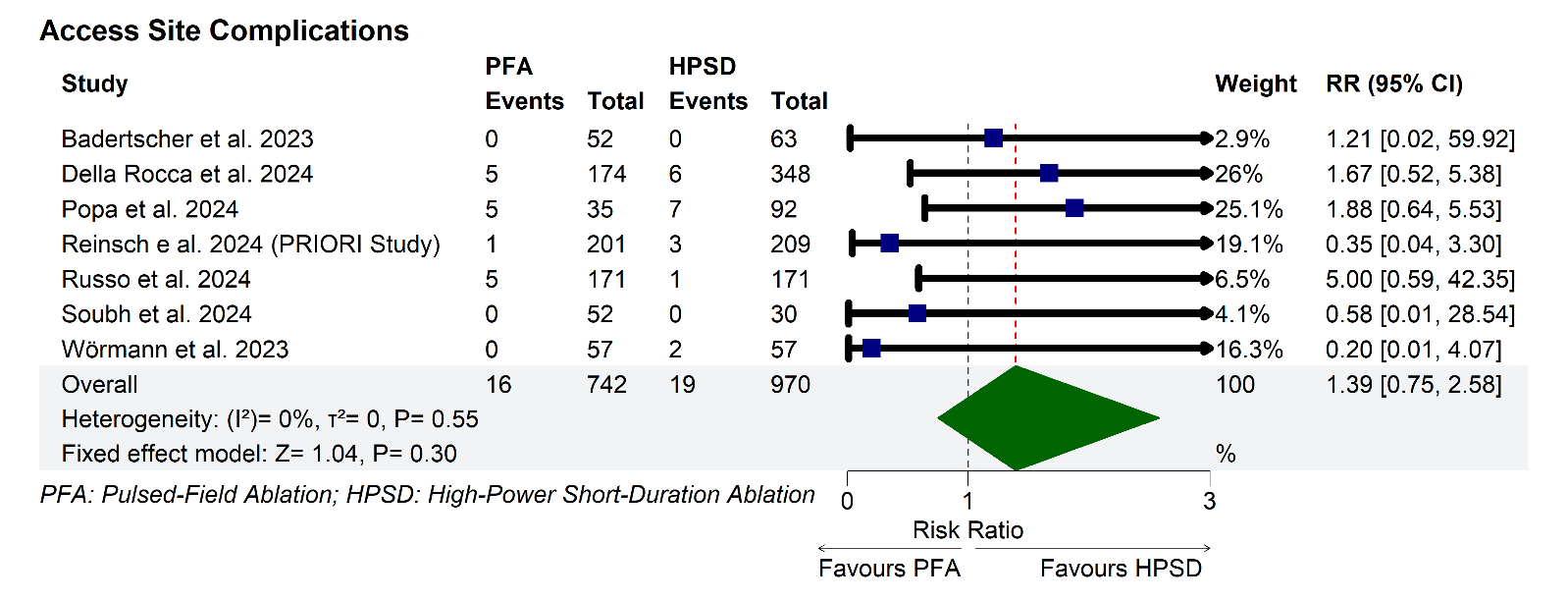


**Figure S27: Forest plot of access site complications.**


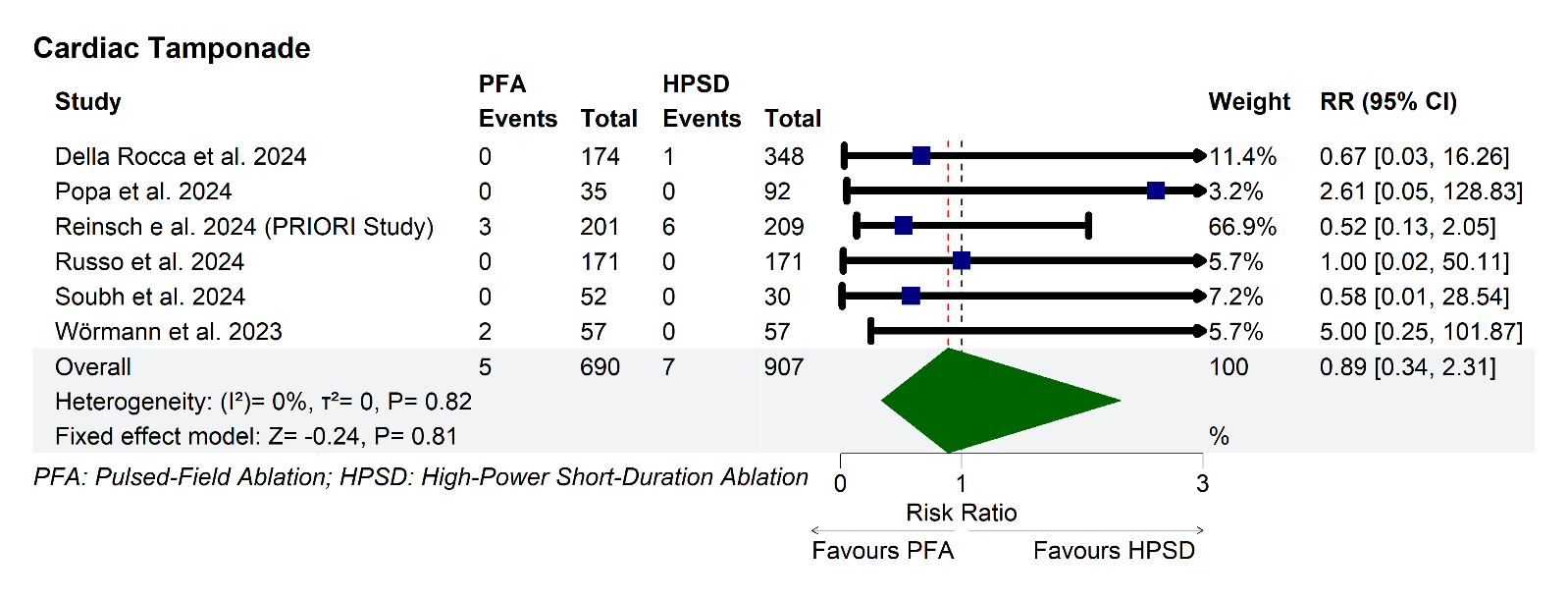


**Figure S28: Forest plot of cardiac tamponade.**


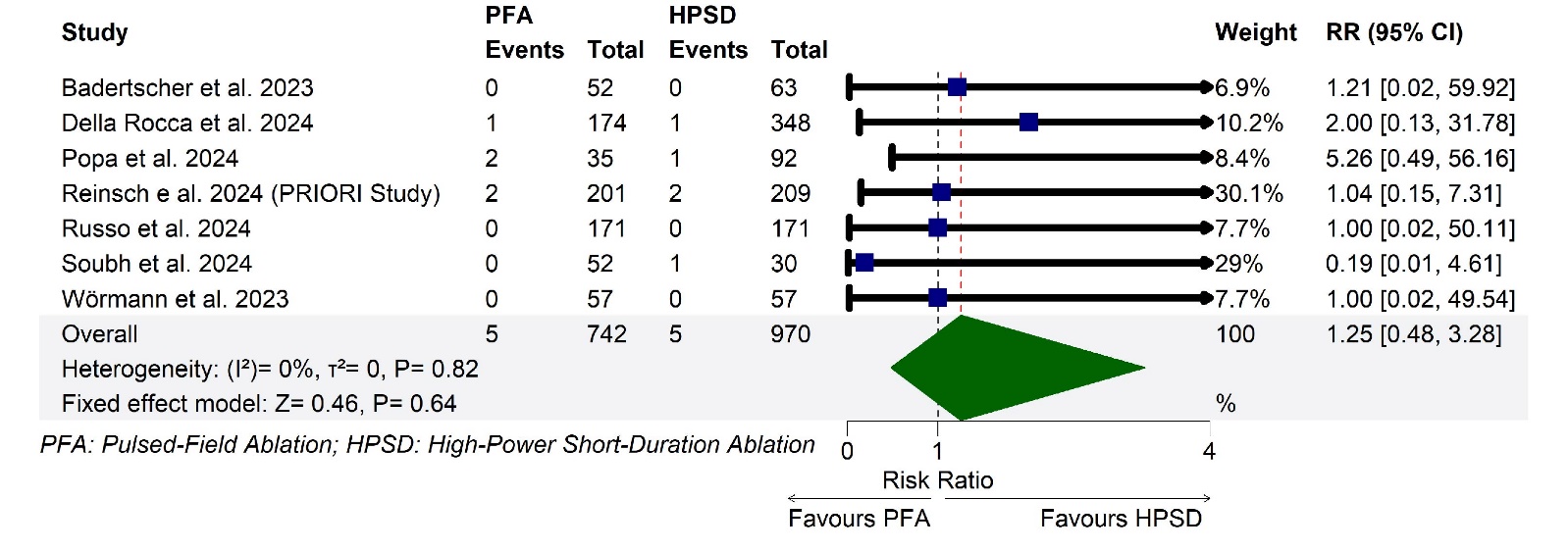


**Figure S29: Forest plot of stroke/TIA.**

**
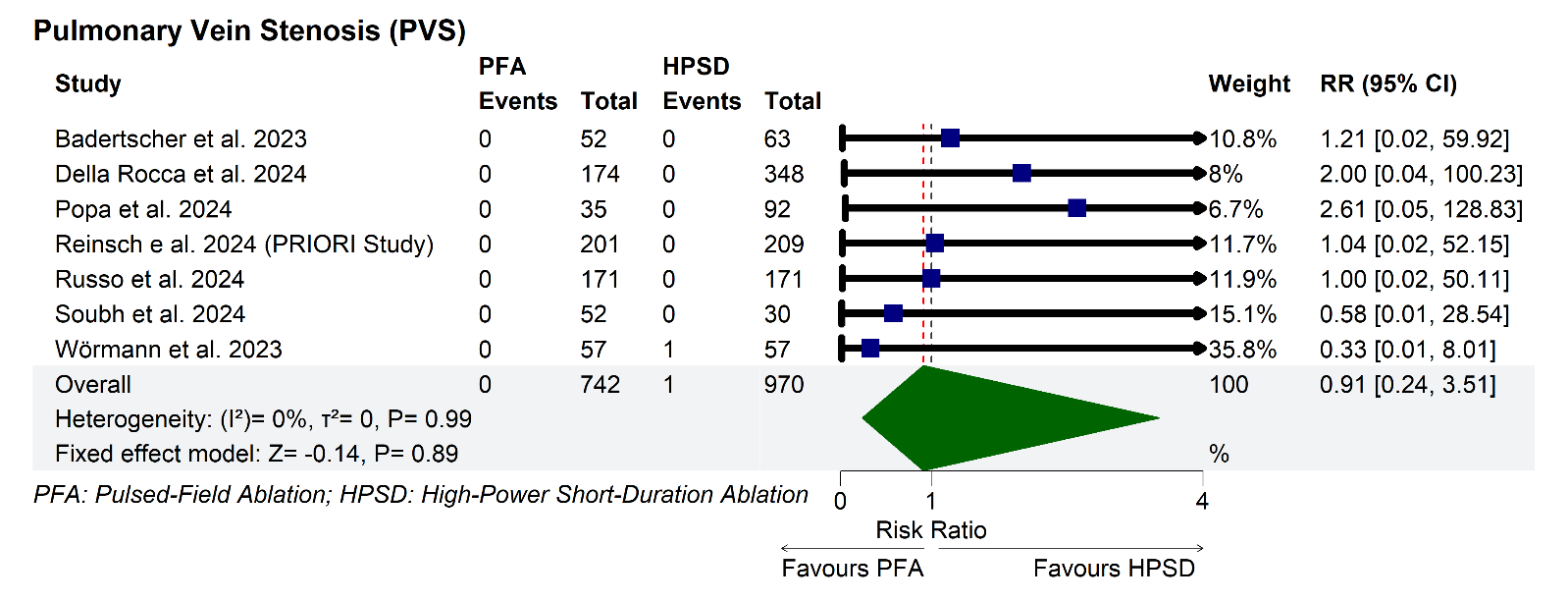
**

**Figure S30: Forest plot of pulmonary vein stenosis.**


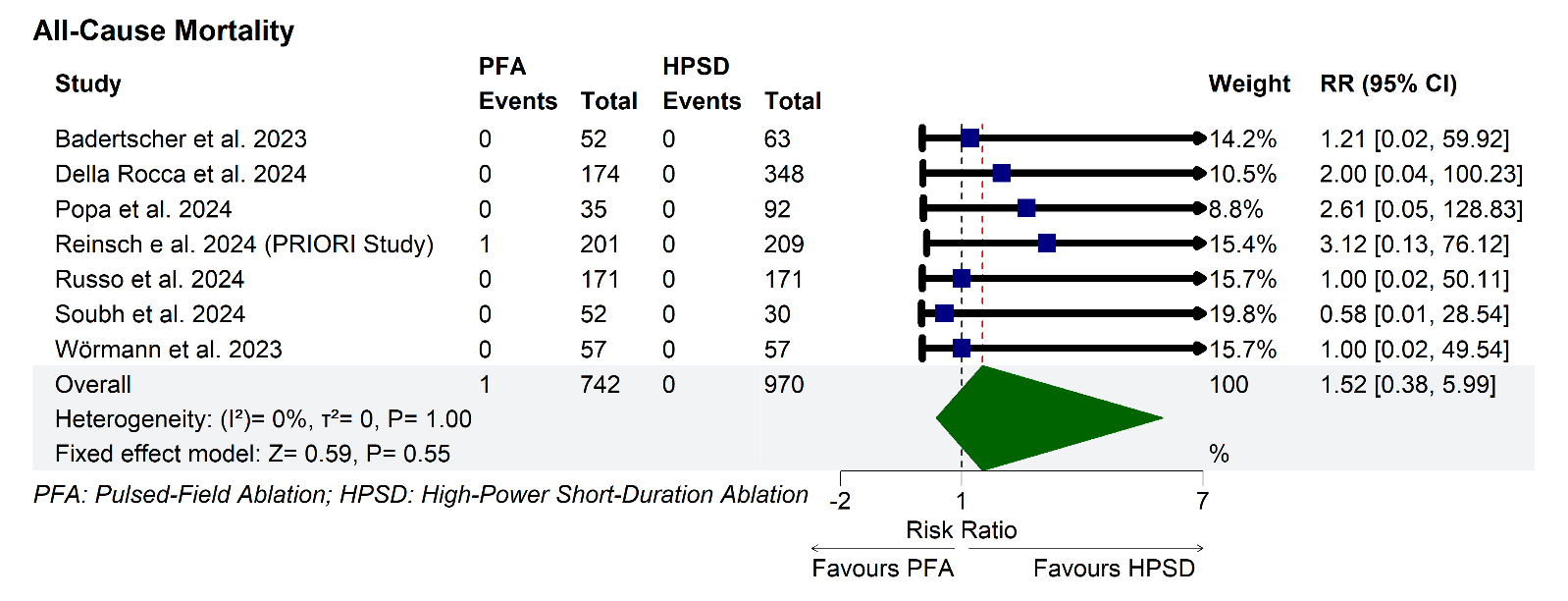


**Figure S31: Forest plot of all-cause mortality.**

**

**

**Figure S32: Subgroup analysis of any adverse events at different power levels.**


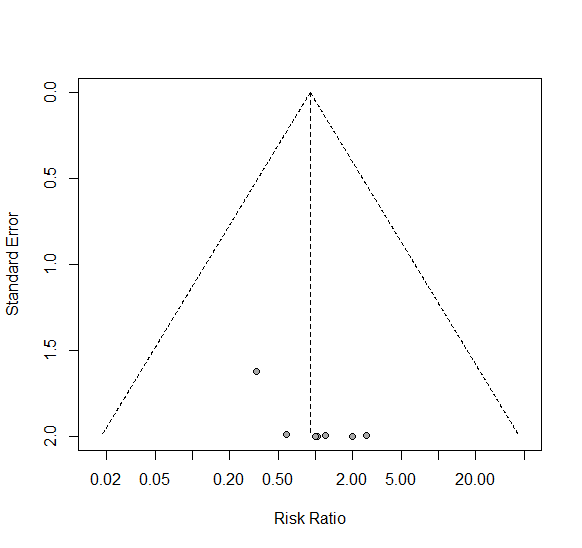


***Figure S33: Funnel plot of Pulmonary vein stenosis.***


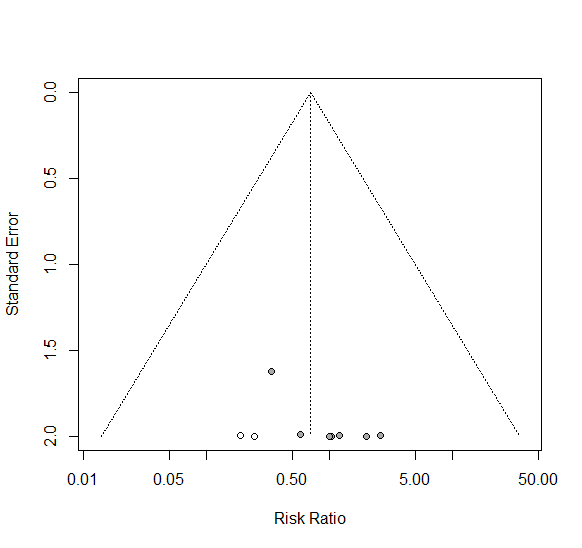


***Figure S34: Trim and fill plot of Pulmonary vein stenosis.***


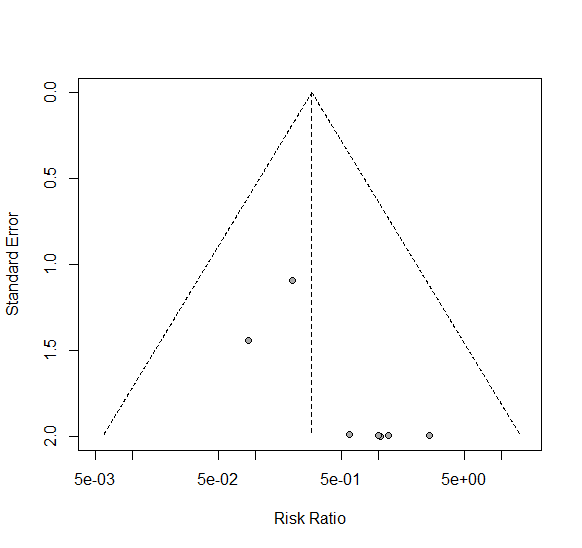


***Figure S35: Funnel plot of pericarditis.***


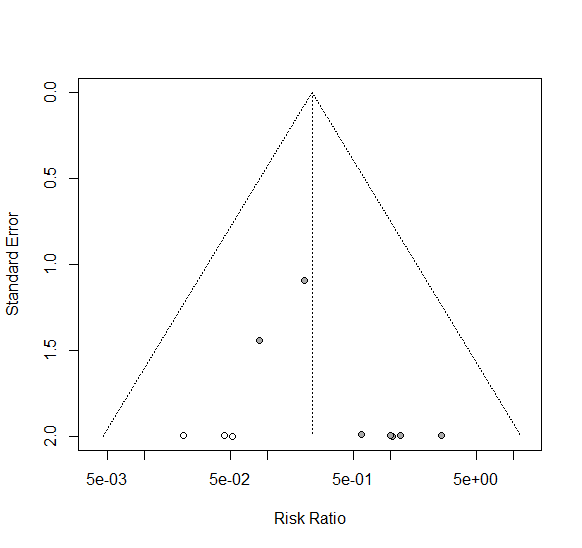


***Figure S36: Trim and fill plot of Pericarditis.***


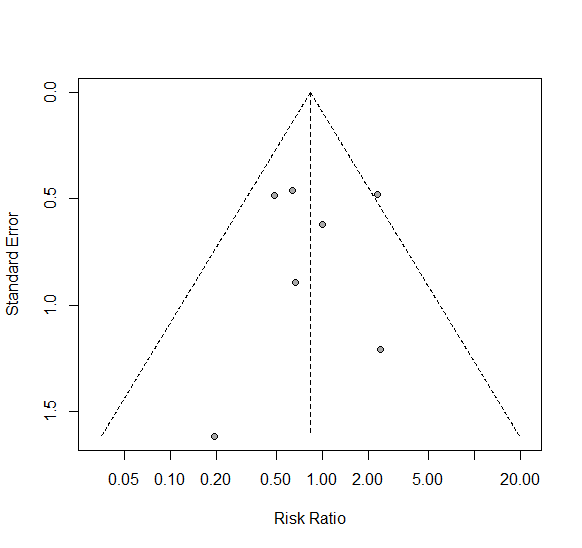


***Figure S37: Funnel plot of any adverse events.***


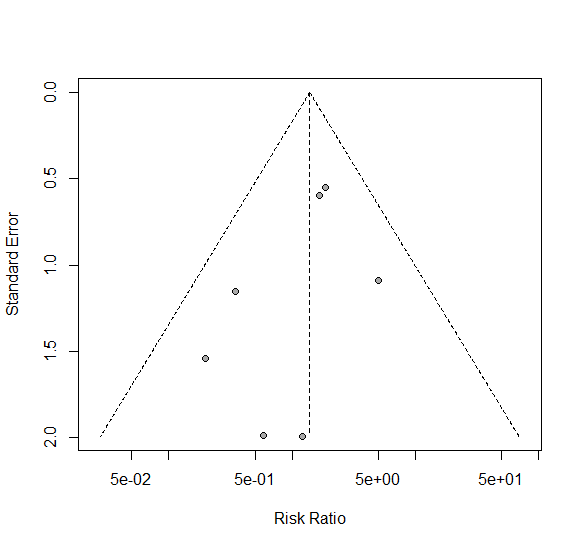


***Figure S38: Funnel plot of access site complications.***

***
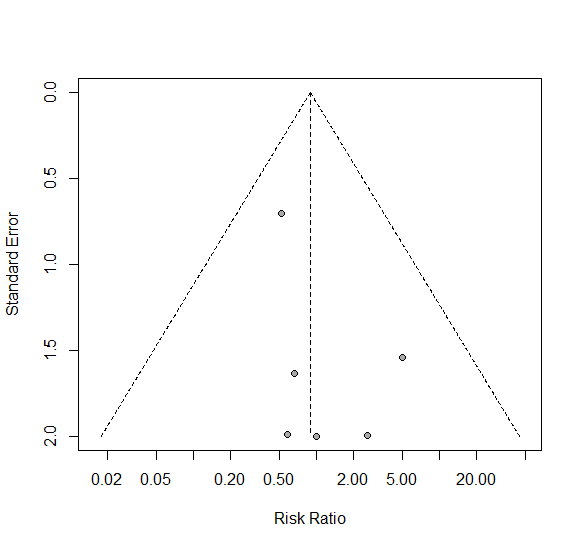
***

***Figure S39: Funnel plot of cardiac tamponade.***

***
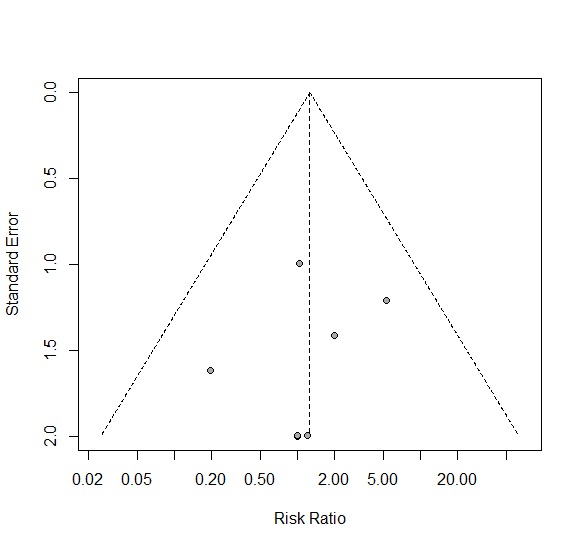
***

***Figure S40: Funnel plot of stroke.***

***
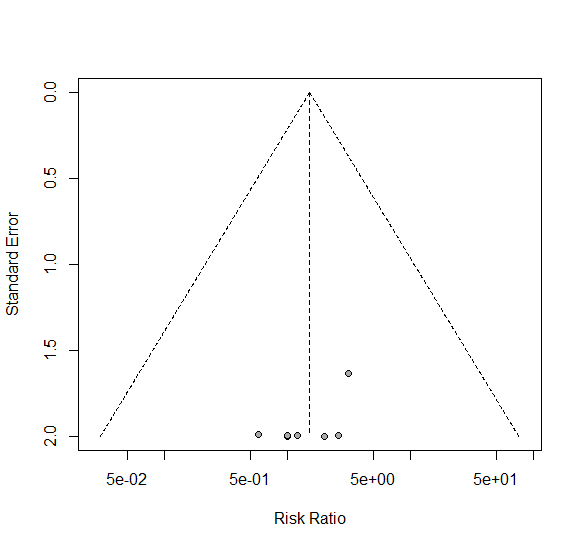
***

***Figure S41: Funnel plot of all-cause mortality.***

| **Database** | **Search Terms** | **Search Field** | **Search Results** |
| --- | --- | --- | --- |
| Pubmed | ("pulsed-field" OR "pulsed field") AND ("High power*" OR "High-power*" OR "High-power ablation" OR "High-power shorter duration" OR HPSD) AND ("atrial fibrillation" OR AF OR afib OR "Persistent Atrial Fibrillation" OR "Paroxysmal Atrial Fibrillation" OR "Auricular Fibrillation") | All Field | 13 |
| Cochrane | ("pulsed-field" OR "pulsed field") AND ("High power*" OR "High-power*" OR "High-power ablation" OR "High-power shorter duration" OR HPSD) AND ("atrial fibrillation" OR AF OR afib OR "Persistent Atrial Fibrillation" OR "Paroxysmal Atrial Fibrillation" OR "Auricular Fibrillation") | All Field | 1 |
| WOS | ("pulsed-field" OR "pulsed field") AND ("High power*" OR "High-power*" OR "High-power ablation" OR "High-power shorter duration" OR HPSD) AND ("atrial fibrillation" OR AF OR afib OR "Persistent Atrial Fibrillation" OR "Paroxysmal Atrial Fibrillation" OR "Auricular Fibrillation") | All Field | 18 |
| SCOPUS | TITLE-ABS-KEY ( ( "pulsed-field" OR "pulsed field" ) AND ( "High power*" OR "High-power*" OR "High-power ablation" OR "High-power shorter duration" OR hpsd ) AND ( "atrial fibrillation" OR af OR afib OR "Persistent Atrial Fibrillation" OR "Paroxysmal Atrial Fibrillation" OR "Auricular Fibrillation" ) ) | Title, Abstract, Keywords | 14 |
| EMBASE | #4. #1 AND #2 AND #3 35  #3. af:ti,ab,kw OR afib:ti,ab,kw OR 'persistent 213,916  atrial fibrillation':ti,ab,kw OR 'paroxysmal  atrial fibrillation':ti,ab,kw OR 'atrial  fibrillation':ti,ab,kw  #2. 'high power*':ti,ab,kw OR 'high-power 24,517  ablation':ti,ab,kw OR 'high-power shorter  duration':ti,ab,kw OR hpsd:ti,ab,kw OR 'high  power short duration ablation':ti,ab,kw OR 'high  power short duration radiofrequency  ablation':ti,ab,kw  #1. 'pulsed field':ti,ab,kw OR 'pulsed field 18,704  ablation':ti,ab,kw | All Field | 35 |

**Table S1: Search Strategy.**

| **Study** | **Pulsed Field Ablation** | | | |
| --- | --- | --- | --- | --- |
|  | **How access** | **Sheath used** | **Mapping used** | **Technique** |
| **Badertscher et al. 2023** | Single transseptal puncture. | No sheath was used. | PVI was assessed by bipolar voltage mapping using a multipolar mapping catheter (PentaRay, Biosense Webster). | 31-mm FaraWave catheter (Boston Scientific, Marlborough, MA) was inserted via a single transseptal puncture. |
| **Della Rocca et al. 2024** | Two ultrasound-guided right groin accesses were used to achieve transseptal access via an 8.5 F SL0 fixed sheath (Abbott, St Paul, MN, USA) and advance a 10-pole catheter into the coronary sinus (CS) or the right ventricle to be used for pacing in case of PFA-related bradycardia. | 8.5 F SL0 fixed sheath (Abbott, St Paul, MN, USA); once transseptal access was achieved, the 8.5 F SL0 fixed sheath was exchanged with the Faradrive™ sheath | No mapping catheter | PFA catheter Farawave™, an over-the-wire device characterized by five splines and 20 electrodes, which can be deployed into two different configurations (basket and flower). At least four pairs of PFA applications (two pairs in basket and two pairs in flower configuration, each pair at ∼36° rotation from the other) were performed to achieve PVI. A complete PFA application set for the segmental superior and inferior was delivered in the case of a common PV ostium. |
| **Popa et al. 2024** | PFA ablation was performed using a single access. After transseptal puncture, the Swartz™ Braided LAMP™ 45 sheath was replaced by the FaraDrive™ sheath (Farapulse), and the pentaspline Farawave™ catheter (Farapulse) was advanced in the LA over a straight 0.035″ stiff guidewire. | After transseptal puncture, the Swartz™ Braided LAMP™ 45 sheath was replaced by the FaraDrive™ sheath (Farapulse) | No mapping catheter | Atropine (0.5–1mg) was administered intravenously before the first pulse to prevent vagal reactions. Intermittent RV pacing was performed with a quadripolar catheter (Supreme, St. Jude Medical) placed in the RV apex in case of vagal reactions involving transient asystole. For each PV, they applied multiple biphasic and bipolar pulse cycles of 2kV with the Farastar™ PFA generator (Farapulse). Specifically, four pulse cycles were used in the “basket” configuration and four in the “flower” configuration. After two consecutive cycles in the same position, the catheter was slightly rotated before applying the two following cycles. Additional pulses were only used in case elimination of all PV potentials by 4+4 pulse cycles was not achieved. The entrance block was confirmed at the end of the procedure by placing the Farawave™ catheter in each PV ostium. |
| **Reinsch e al. 2024 (PRIORI Study)** | After a double right femoral vein puncture, a decapolar electrode catheter (Webster CS, Biosense Webster, Irvine, CA, USA) was inserted in the coronary sinus. Under fluoroscopic guidance, a single transseptal puncture was performed using an 8F-sheath (SR0 Fast-Cath Guiding Introducer, St. Jude Medical, Minneapolis, MN, USA). After transseptal puncture, the 8F-sheath was exchanged for a 13F inner diameter, 16.8F outer diameter transparent sheath (Faradrive, Farapulse, Menlo Park, CA, USA). The sheath was continuously irrigated with heparinized saline. Initially, a 0.035-inch, 180-cm extra-stiff, straight-tip guidewire (Amplatz Extra Stiff, Cook Medical, Bloomington, IN, USA) was used as a rail to deploy the multielectrode 12F over the-wire PFA catheter (Farawave, Farapulse, Menlo Park, CA, USA) into the desired shape and advanced it into position at the antrum of each PV. A J-tip guidewire replaced The straight guidewire during the study period (InQwire, Merit Medical System, Inc, South Jordan, UT, USA). | Under fluoroscopic guidance, a single transseptal puncture was performed using an 8F-sheath (SR0 Fast-Cath Guiding Introducer, St. Jude Medical, Minneapolis, MN, USA). After transseptal puncture, the 8F-sheath was exchanged for a 13F inner diameter, 16.8F outer diameter transparent sheath (Faradrive, Farapulse, Menlo Park, CA, USA). | Carto SEG (Biosense Webster) software module, the 3D left atrial and pulmonary vein reconstruction of the CT angiography, was used as a roadmap to position the guidewire and the PFA catheter fluoroscopically. | At full deployment, two catheter sizes were available: 31 and 35 mm in maximal diameter. The choice of catheter size depended on the maximal PV diameter in the left atrial CT angiography. The case for a PV > 27mm diameter from superior to inferior, the 35mm PFA was chosen. Ablative energy was delivered from all electrodes, recording electrograms, and pacing was possible through the third electrode of each spline. Ablation procedural workflow was described in detail previously. In brief, eight biphasic pulse trains with a power of 1900 or 2000 V were applied per PV, four applications each in the basket/biscuit and flower poses. Between the first pair of applications and the second pair of applications in a particular pose, the over-the-wire catheter was rotated 30–40°. Using the Carto SEG (Biosense Webster) software module, the 3D left atrial and pulmonary vein reconstruction of the CT angiography was used as a roadmap to position the guidewire and the PFA catheter fluoroscopically. Selective PV angiographies and biplane fluoroscopy were used to optimize contact between the PFA catheter and tissue and ensure adequate coverage of the PV antra for all patients. |
| **Russo et al. 2024** | After two ultrasound-guided right femoral vein accesses were retrieved, a steerable decapolar catheter was advanced into the coronary sinus, and transseptal puncture was performed using an 8.5Fr SL0 fixed sheath (Abbott, MN, USA) and a Brockenbrough needle. After transseptal puncture, the SL0 sheath was exchanged with the Faradrive (Boston Scientific, USA) steerable sheath using a guidewire, and the 31-mm Farawave ablation catheter was subsequently advanced into PVs’ antra over the wire. | The transseptal puncture was performed using an 8.5Fr SL0 fixed sheath (Abbott, MN, USA) and a Brockenbrough needle. After transseptal puncture, the SL0 sheath was exchanged with the Faradrive (Boston Scientific, USA) steerable sheath using a guidewire. | A steerable decapolar catheter was advanced into the coronary sinus. | A 1-mg atropine bolus was delivered to mitigate PFA-induced bradycardia. The Farawave catheter is a 5-spline, 20 electrodes, basket/flower configuration catheter. Per each vein, 8 PFA applications were delivered, 4 in basket and 4 in flower configurations; after the first two applications in each configuration, the catheter was rotated by 30°/45° to cover the entire pulmonary vein (PV) circumference. In patients with persistent atrial fibrillation (AF) and risk factors for AF recurrence (i.e., left atrial [LA] dilation and/or long duration of persistent AF episode), and/or in patients with LA low-voltage areas and/or in patients undergoing redo ablation, LA posterior wall (LA-PW) ablation was also performed, using the PFA catheter in the flower configuration, with the wire retracted, and delivering 2 PFA applications per each LA-PW location, in an overlapping fashion across the entire LA-PW.4 PVI and LA-PW isolation were validated at the end of procedure by demonstrating entrance and exit PV/LA-PW block 20min after the last PFA application in each PV and/or in the LA-PW, as follows: successful PV entrance block was confirmed by demonstrating absence of near-field electrograms after repositioning the Farawave catheter in the basket configuration into each PV antrum, while successful PV exit block was verified by demonstrating lack of LA capture by pacing (10 mA/2 msec) from the Farawave catheter into each PV antrum; successful LA-PW entrance block was defined by the absence of near-field electrograms within the endocardial surface of the LA-PW, while successful LA-PW exit block was verified by demonstrating lack of LA capture by pacing (10 mA/2 msec) from within the LA-PW. Touch-up PFA applications were delivered until LA-PW isolation was achieved. |
| **Soubh et al. 2024** | two sheaths (7 French) were inserted into the left femoral vein, and one sheath (8 French) was inserted into the right femoral vein. A diagnostic decapolar catheter (Inquiry, Abbott, USA) was positioned in the coronary sinus. The transseptal puncture (TSP) with the Brockenbrough needle (BRK-1, Abbott, USA) was performed under fluoroscopic and pressure guidance using a non-steerable sheath (Fast-Cath SL1, 8,5 French, Abbott, USA). Fluoroscopy with two pulses per second was used for catheter placement and TSP. A contrast agent was applied for selective pulmonary vein angiography in all procedures. After selective pulmonary vein angiography, an anatomical and voltage mapping in the left atrium was performed using a 3D mapping system (Ensite X, Abbott, USA, or CARTO® 3 System, Biosense Webster, USA) and a mapping catheter, the Advisor™ HD Grid catheter (Abbott, USA) or the Pentaray™ NAV ECO (Biosense Webster, USA). After that, the SL1 sheath was replaced by the steerable PFA sheath (Faradrive, Boston Scientific, USA). | Two sheaths (7 French) were inserted into the left femoral vein, and one sheath (8 French) was inserted into the right femoral vein. | A diagnostic decapolar catheter (Inquiry, Abbott, USA) was positioned in the coronary sinus. An anatomical and voltage mapping in the left atrium was performed using a 3D mapping system (Ensite X, Abbott, USA, or CARTO® 3 System, Biosense Webster, USA) and a mapping catheter, the Advisor™ HD Grid catheter (Abbott, USA) or the Pentaray™ NAV ECO (Biosense Webster, USA). | Pulsed-field energy was applied eight times per pulmonary vein at ostial positions, four times in the “basket” configuration, and four times in the “flower” configuration. At the end of the procedure, an LA re-map was performed to visualize the ablation lesions and ensure complete isolation of all veins. |
| **Wörmann et al. 2023** | Double (PFA) groin access was established via the right femoral vein. A decapolar catheter (Dynamic XT™, large curve 4.0/Decapolar; Boston Scientific) was positioned in the coronary sinus (CS). Transseptal puncture (TSP) was performed using TSX™ fixed curve transseptal sheath and TSX™ transseptal needle (Boston Scientific). | Transseptal puncture (TSP) was performed using TSX™ fixed curve transseptal sheath and TSX™ transseptal needle (Boston Scientific). | A decapolar catheter (Dynamic XT™, large curve 4.0/Decapolar; Boston Scientific) was positioned in the coronary sinus (CS). In the first 14 (25%) procedures, an electroanatomic map (EAM) of the left atrium (LA) was acquired with a multipolar mapping catheter (Pentaray™; Biosense Webster or HD‐Grid; Abbott Abbott Park) after treatment of all PVs to verify the entrance block. In the following procedures, proof of PV isolation was obtained by abolishing local PV electrograms obtained by the FARAWAVE™catheter. | VAll PFA procedures were performed with the FARAPULSE™ system (Boston Scientific). After TSP, the 8,5‐F TSX sheath was replaced by the 13‐Fsheath (FARADRIVE™; Boston Scientific). In the first 30 procedures, a straight guidewire (Amplatz extra stiff straight wire™; Cook Group Incorporated) was used to intubate the PVs. Over the wire, the PFA catheter (FARAWAVE™; Boston Scientific) was then positioned at the respective PV antrum. After 30 procedures, a J‐tip guidewire (InQWire™; Merit Medical) was used instead of the straight guidewire as recommended by the manufacturer. At every PV antrum, 8 PFA impulses were delivered in the “flower” and “basket” configuration of the catheter. The delivery of more PFA applications was at the operator's discretion. The FARAWAVETM catheter was used to display the local electrograms at the PV antrum (before and between the PFA impulses) and inside the PV (after the impulses) to reveal the entrance block. In the first 14 (25%) procedures, an electroanatomic map (EAM) of the left atrium (LA) was acquired with a multipolar mapping catheter (Pentaray™; Biosense Webster or HD‐Grid; Abbott Abbott Park) after treatment of all PVs to verify the entrance block. In the following procedures, proof of PV isolation was obtained by abolishing local PV electrograms obtained by the FARAWAVE™catheter. |

***Table S2: PFA Procedure details across the studies.***

| **Study** | **High-power short duration ablation** | | | |
| --- | --- | --- | --- | --- |
|  | **How access** | **Sheath used** | **Mapping used** | **Technique** |
| **Badertscher et al. 2023** | Using a single transseptal puncture | No sheath was used. | Without any multipolar mapping catheter. | PVI was performed with an irrigated-tip ThermoCool catheter at 50 W (Smarttouch SF, Biosense Webster, Irvine, CA). |
| **Della Rocca et al. 2024** | Three right groin accesses under ultrasound guidance were used to achieve double transseptal access via an 8.5 F SL0 fixed sheath (Abbott, St Paul, MN, USA) and advance a 10-pole catheter into the CS. | 8.5 F SL0 fixed sheath (Abbott, St Paul, MN, USA) | A 3D-electroanatomical mapping system (Carto 3, Biosense Webster, Diamond Bar, CA, USA) guided mapping and ablation. | Left atrial electroanatomical mapping and broad antral circumferential ablation (WACA) were achieved via a multipolar catheter (Pentaray) and an open-irrigated RF ablation catheter (Thermocool SmartTouch Surround Flow, Biosense Webster, Diamond Bar, CA, USA). Radiofrequency ablation was performed with a power of 45 W, a desired contact force of 10–15 g, and a target ablation index of 500 for anterior and 400–450 for posterior PV segments. The oesophageal temperature was monitored throughout the procedure via a multi-sensor Circa S-Cath™ oesophageal temperature probe (Circa, Scientific Inc., CO, USA). |
| **Popa et al. 2024** | Double access to the left atrium (LA) was obtained. Fluoroscopy‐guided single transseptal puncture was performed using the Agilis™ steerable sheath (Abbott) in the HPSD 70W group. The CARTO VIZIGO™ (Biosense Webster) sheath in the HPSD‐90W group. | Fluoroscopy‐guided single transseptal puncture was performed using the Agilis™ steerable sheath (Abbott) in the HPSD 70W group. The CARTO VIZIGO™ (Biosense Webster) sheath in the HPSD‐90W group. | High‐density electroanatomical mapping was performed using EnSite™ Precision and the Advisor circular catheter (Abbott) in HPSD‐70W. The CARTO® 3 system and the Lasso catheter (BiosenseWebster) were employed in HPSD‐90W. | Wide antral circumferential PVI was performed using point‐by point‐lesions with the 4mm irrigated‐tip catheter FlexAbility™ SE and the Ampere® RFgenerator (Abbott; standard RFA and HPSD 70W) or the 3.5mm irrigated‐tip catheter QDOT MICRO™ and the nGEN™ RF generator (Biosense Webster; HPSD‐90W). HPSD‐70W group: 70W/7s (anterior wall) or 70W/5s (posterior wall) were applied. Ablation was performed using a power-controlled mode (automatic temperature cut‐off of 42°C and 20mL/min [HPSD‐70W]) and an automated duration control limited RF delivery. An interlesion distance of 5–6mm (HPSD‐70W) was targeted. HPSD‐90W group: 90W/4s was applied on the anterior and posterior wall in a temperature‐controlled mode (QMODE+) with a target temperature of 60°C. If first‐pass PVI was not achieved, touch‐up RF lesions using 50W/15s were applied in the conventional QMODE. An interlesion distance of 5–6mm was targeted. |
| **Reinsch e al. 2024 (PRIORI Study)** | After a triple right femoral vein puncture, a decapolar electrode catheter (Webster CS, Biosense Webster, Irvine, CA, USA) was inserted in the coronary sinus. Under fluoroscopic guidance, we performed a double transseptal puncture, directlynch (F) sheaths (SL1 Fast-Cath Guiding Introducer, St. Jude Medical, Minneapolis, MN, USA), directly after the transseptal puncture. The sheaths were continuously irrigated with heparinized saline. After transseptal access, a 3D electroanatomic map (EAM) of the left atrium and pulmonary veins (PVs) was constructed using a non-fluoroscopic navigation system (Carto 3®, Biosense Webster Inc., Diamond Bar, CA, USA) and pentaspline mapping catheter (Pentaray, Biosense Webster, Diamond Bar, CA, USA). Fast anatomic mapping (FAM) was performed in all patients. | After transseptal access, a 3D electroanatomic map (EAM) of the left atrium and pulmonary veins (PVs) was constructed using a non-fluoroscopic navigation system (Carto 3®, Biosense Webster Inc., Diamond Bar, CA, USA) and pentaspline mapping catheter (Pentaray, Biosense Webster, Diamond Bar, CA, USA). Fast anatomic mapping (FAM) was performed in all patients. | A decapolar electrode catheter (Webster CS, Biosense Webster, Irvine, CA, USA) was inserted in the coronary sinus. After transseptal access, a 3D electroanatomic map (EAM) of the left atrium and pulmonary veins (PVs) was constructed using a non-fluoroscopic navigation system (Carto 3®, Biosense Webster Inc., Diamond Bar, CA, USA) and pentaspline mapping catheter (Pentaray, Biosense Webster, Diamond Bar, CA, USA). Fast anatomic mapping (FAM) was performed in all patients. | In power control mode, RF applications were delivered using a 3.5-mm Thermocool Smarttouch SF® Catheter (Biosense Webster, Inc., Diamond Bar, CA, USA). RF power was set to 45 W, and the catheter tip was irrigated by saline at a flow rate of 2 mL/min during mapping and 15 mL/min during ablation, respectively. Ablation was performed by adhering to the CLOSE protocol. RF energy was delivered until an Ablation Index (AI) reached 450 at the posterior wall/ inferior/ roof and 600 at the anterior wall. The continuity of both ablation circles was further checked using complete transparency of the voltage map. Upon completion of circumferential ablation, the pentaspline mapping catheter was used to demonstrate both entrance- and exit-block to confirm bidirectional PV isolation. In the absence of isolation after completion of the circle, touch-up ablation was delivered until bidirectional PV isolation was achieved. |
| **Russo et al. 2024** | Three right femoral vein ultrasound-guided accesses were retrieved and used to advance a decapolar steerable catheter into the coronary sinus, perform a single transseptal puncture (using an 8.5Fr SL0 fixed sheath and a Brockenrough needle), and advance QDOT Micro (QDM) and a multielectrode mapping catheter into the LA. High-density electroanatomical maps of the LA were reconstructed using fast anatomical mapping, and antral PVI was performed with point-by-point RF applications in the PV antra and the intervenous carina. | Using an 8.5Fr SL0 fixed sheath and a Brockenrough needle. | A decapolar steerable catheter into the coronary sinus, a multielectrode mapping catheter into the LA. High-density electroanatomical maps of the LA were reconstructed using fast anatomical mapping. | An esophageal thermal probe was inserted at the beginning of the procedure. In posterior segments of the PVs, vHPSD RF applications (QMODE+, 90Watts/4sec, 8 mL/min flow rate; ablation stopped automatically if the temperature increased above the 65ºC cutoff) were delivered, and the recommended intervention distance was 6 mm. In anterior segments, operators either used the vHPSD mode, with interlesion distance of 4mm (pure vHPSD), or temperature-controlled 50Watts pulses (QMODE) targeting AI values of 500-550 (hybrid vHPSD). RF applications were started after a stable catheter position was obtained, with contact force ≥5g. Ablation was interrupted as soon as the esophageal temperature reached 39°C, and further RF was not delivered on posterior LA regions until the esophageal temperature returned to baseline level. The ablation of additional LA structures (LA-PW, LA appendage, coronary sinus [CS]) was performed using vHPSD (for LA-PW and LAA) or temperature-controlled ablation (in the CS); procedural details are reported in the following paragraph. Twenty minutes after the end of PVI, persistent bidirectional PVI was confirmed by demonstrating PV entrance block via remapping PV antra using a multielectrode catheter and PV exit block by pacing with 10mA@2 msec pulses from within the PV antra. For PV touch-ups, operators used the vHPSD mode. |
| **Soubh et al. 2024** | Two sheaths (7 French) were inserted into the left femoral vein, and one sheath (8 French) was inserted into the right femoral vein. A diagnostic decapolar catheter (Inquiry, Abbott, USA) was positioned in the coronary sinus. The transseptal puncture (TSP) with the Brockenbrough needle (BRK-1, Abbott, USA) was performed under fluoroscopic and pressure guidance using a steerable sheath (CARTO Vizigo, Biosense Webster, USA). Fluoroscopy with two pulses per second was used for catheter placement and TSP. A contrast agent was applied for selective pulmonary vein angiography in all procedures. After selective pulmonary vein angiography, a high-density anatomical and voltage mapping in the left atrium was performed using the PENTARAY® NAV ECO mapping catheter and the CARTO® 3 System (Biosense Webster, USA). | a steerable sheath (CARTO Vizigo, Biosense Webster, USA) | A diagnostic decapolar catheter (Inquiry, Abbott, USA) was positioned in the coronary sinus. A high-density anatomical and voltage mapping in the left atrium was performed using the PENTARAY® NAV ECO mapping catheter and the CARTO® 3 System (Biosense Webster, USA). | After the pulmonary vein ostia were marked, circumferential lesions were created employing the QDOT MICRO™ catheter (Biosense Webster, USA) and the QMode + (90 watts for 4 s, targeted interlesion distance 4 mm) |
| **Wörmann et al. 2023** | Triple (VHPSD) groin access was established via the right femoral vein. A decapolar catheter (Dynamic XT™, large curve 4.0/Decapolar; Boston Scientific) was positioned in the coronary sinus (CS). Transseptal puncture (TSP) was performed using TSX™ fixed curve transseptal sheath and TSX™ transseptal needle (Boston Scientific). | Transseptal puncture (TSP) was performed using TSX™ fixed curve transseptal sheath and TSX™ transseptal needle (Boston Scientific). | A decapolar catheter (Dynamic XT™, large curve 4.0/Decapolar; Boston Scientific) was positioned in the coronary sinus (CS). Circumferential PVI (entrance block) was monitored with the circumferential mapping catheter. | Esophageal temperature was monitored using a temperature probe (S‐Cath, Esophageal Temperature Probe; Circa Scientific Inc.). Ablation was stopped when esophageal‐probe temperatures exceeded >41°C to prevent atrial esophageal fistula formation or erosion. All VHPSD procedures were performed using an EAM system (EnsitePrecision™ or Ensite X™; Abbott). All LA‐EAM were acquired using a circumferential decapolar catheter (Advisor FL™ Sensor Enabled; Abbott). A noncontact‐force ablation catheter with enhanced tip irrigation (20 mL/min) and distally positioned thermocouples (Flexibility D‐ or F‐Curve; Abbott) was used due to its favorable design for vHPSDablation and thermal tissue conduction and measurement. Antral PVI was achieved using point‐by‐point ablation with the isolation of both PV pairs to avoid overlap of the ablation lesion projections. For circumferential PVI, a power setting of 70W for 7s was used at all sites except for the posterior wall, where RF duration was reduced to 5s. Ablation with VHPSD was performed in a power‐controlled mode, which is safe and effective. Time limits were set at 7/5s, and a temperature cut‐off of 42°C was used. Lesion projections were set to 5mm since lesion width is reported to range from 5 to 11mm in in vitro studies for VHPSD. Interlesion distance and lesion overlap were avoided. An interlesion‐distance of max. 2mm was accepted, followed by a reassessment using pacing along the ablation line with the non‐excitability endpoint. This setting uses an enhanced irrigated tip catheter and showed the most favorable outcomes regarding arrhythmia‐free survival and procedure duration and was, therefore, the comparator in this study. |

***Table S3: HPSD ablation Procedure details across the studies.***

| **Study** | **Matching Criteria** | **Covariates** |
| --- | --- | --- |
| **Della Rocca et al. 2024** | Propensity score matching was performed with a 1:2 ratio to reduce the imbalance of covariates among groups.  Matching was performed using the nearest neighbor matching protocol (matching ratio of 1 to 2 without replacement) and a caliper width of 0.01. | Age, gender, hypertension, diabetes, heart failure, coronary/peripheral artery disease, history of thromboembolic events, chronic kidney disease, and left atrial size. |
| **Russo et al. 2024** | Propensity score matching was performed with a 1:1 ratio to control for confounding because of imbalance of covariates between groups. The nearest neighbor method without replacement was used, and the caliper width was set at 0.20. | Age, Male sex, Female sex, Paroxysmal AF, Persistent AF, BMI (Kg/m2), Arterial hypertension, Type 2 Diabetes mellitus, Hx of prior AF ablation, iLAV (ml/m2), CHA_2_DS_2_-VASc score, EHRA symptom class, eGFR (ml/min), Ischemic heart disease, Heart Failure, Moderate/Severe VHD, LVEF (%), Hx of atrial flutter, Class Ic AADs, Class III AADs, Beta blockers, Class IV AADs, MRA, ARNI, SGLT2inh, and Obesity |

***Table S4: Propensity Score Matching Details used in Della Rocca et al. and Russo et al.***

**AF: atrial fibrillation, BMI: Body mass index; AADs: antiarrhythmic drugs; ARNI: angiotensin receptor–neprilysin inhibitor; eGFR: estimated glomerular filtration rate; EHRA: European Heart Rhythm Association; Hx: history; iLAV: indexed left atrial volume; LVEF: left ventricular ejection fraction; MRA: mineralocorticoid receptor antagonist; SGLT2inh: sodium-glucose cotransporter 2 inhibitor; VHD: valvular heart disease.**

| Outcome | Chi-square | p-value |
| --- | --- | --- |
| **Any atrial tachyarrhythmia recurrence** | 30.23 | < 0.001 |

**Table S5: between-study heterogeneity assessment by frailty model.**

| **Excluded study** | **HR** | **Lower bound** | **Upper bound** | **P-value** |
| --- | --- | --- | --- | --- |
| Badertscher et al. 2023 | 0.73 | 0.57 | 0.93 | **0.010** |
| Della Rocca et al. 2024 | 0.72 | 0.54 | 0.96 | **0.027** |
| Reinsch e al. 2024 (PRIORI Study) | 0.73 | 0.57 | 0.95 | **0.017** |
| Russo et al. 2024 | 0.64 | 0.49 | 0.84 | **0.001** |
| Soubh et al. 2024 | 0.74 | 0.59 | 0.94 | **0.014** |
| Wörmann et al. 2023 | 0.75 | 0.59 | 0.96 | **0.020** |

**Table S6: Results from Jackknife sensitivity analysis.**

| **Variable** | **Estimate** | **SE** | **Confidence interval** | **P-value** |
| --- | --- | --- | --- | --- |
| Age, years | -0.0084 | 0.0552 | [ -0.1166, 0.0999] | 0.8796 |
| Male percentage | 0.0023 | 0.0140 | [ -0.0250, 0.0297] | 0.8664 |
| Body mass index | -0.1233 | 0.1192 | [ -0.3569, 0.1104] | 0.3012 |
| Left ventricular ejection fraction | 0.0673 | 0.0696 | [ -0.0690, 0.2037] | 0.3332 |
| Hypertension | 0.0002 | 0.0094 | [ -0.0181, 0.0186] | 0.9799 |
| Diabetes | 0.0266 | 0.0199 | [ -0.0124, 0.0656] | 0.1813 |
| Coronary artery disease | 0.0030 | 0.0199 | [ -0.0360, 0.0420] | 0.8801 |
| Previous stroke/TIA | -0.0436 | 0.0599 | [ -0.1610, 0.0739] | 0.4674 |
| left atrium diameter (LAD) | -0.0635 | 0.2016 | [ -0.4587, 0.3316] | 0.7527 |

**Table S7: Results from meta-regression analysis.**
